# Spectroscopic, X-ray Diffraction and Density Functional Theory Study of Intra- and Intermolecular Hydrogen Bonds in Ortho-(4-tolylsulfonamido)benzamides

**DOI:** 10.3390/molecules26040926

**Published:** 2021-02-10

**Authors:** Malose J. Mphahlele, Eugene E. Onwu, Marole M. Maluleka

**Affiliations:** 1 Department of Chemistry, College of Science, Engineering and Technology, University of South Africa, Private Bag X06, Florida 1710, South Africa; 64061639@mylife.unisa.ac.za; 2 Department of Chemistry, University of Limpopo, Private Bag X1106, Sovenga 0727, South Africa; marole.maluleka@ul.ac.za

**Keywords:** 2-((4-methylphenyl)sulfonamido)benzamides, intramolecular hydrogen bonding, X-ray, Hirshfeld analysis, DFT

## Abstract

The conformations of the title compounds were determined in solution (NMR and UV-Vis spectroscopy) and in the solid state (FT-IR and XRD), complemented with density functional theory (DFT) in the gas phase. The nonequivalence of the amide protons of these compounds due to the hindered rotation of the C(O)–NH_2_ single bond resulted in two distinct resonances of different chemical shift values in the aromatic region of their ^1^H-NMR spectra. Intramolecular hydrogen bonding interactions between the carbonyl oxygen and the sulfonamide hydrogen atom were observed in the solution phase and solid state. XRD confirmed the ability of the amide moiety of this class of compounds to function as a hydrogen bond acceptor to form a six-membered hydrogen bonded ring and a donor simultaneously to form intermolecular hydrogen bonded complexes of the type N–H···O=S. The distorted tetrahedral geometry of the sulfur atom resulted in a deviation of the sulfonamide moiety from co-planarity of the anthranilamide scaffold, and this geometry enabled oxygen atoms to form hydrogen bonds in higher dimensions.

## 1. Introduction

Sulfonamides are an important class of therapeutic agents, with a broad range of pharmacological profiles, and many sulfonamide-based compounds are in clinical use as active pharmaceutical ingredients for the treatment of bacterial and viral infections, hyperglycemia and hypertension, among others [1,2]. Although predominantly employed as antibacterial agents, sulfonamide-based compounds also exhibit inhibitory properties against various enzymes, including carbonic anhydrase, cysteine protease, HIV protease, cyclooxygenase, acetylcholinesterase (AChE) and butyrylcholinestearse (BChE) [2]. Their enzyme inhibitory properties result from the propensity of the sulfonamide group to establish strong electrostatic (π-cation, attractive charge interaction) and hydrogen bonding interactions with a wide range of functional groups of protein residues. Interest in sulfonamides is not only limited to drug development, but also exists in terms of their conformations and crystalline structures [3,4,5]. This scaffold provides a versatile template to explore hydrogen bonding interactions, control molecular conformations and improve the physicochemical properties of the drug molecules [6,7]. The sulfonamide group generally adopts tetrahedral geometry, and the oxygen atoms of this group easily form hydrogen bonds in higher dimensions than amides do. Aromatic sulfonamides have been found to display conformational chirality emanating from the synclinal conformation [6]. The *N*-phenylbenzenesulfonamides, for example, exist preferentially in (+)- or (−)-synclinal conformations with a twist in the same direction of the aromatic rings at both ends. The hydrogen atom of the sulfonamide group was found to be involved in an intermolecular hydrogen bonding interaction with the O atom of the sulfonyl group of another, which is generally stronger than the other intermolecular interactions, such as CH–π, CH–lone pair, aromatic–aromatic, and other hydrogen bonding interactions. Kikkawa et al. classified the intermolecular hydrogen-bonding patterns of aromatic sulfonamides into four types, namely, dimeric, zigzag, helical, and straight patterns, all retaining the synclinal conformation of the sulfonamide functionality [6]. Integrating intramolecular hydrogen bonding formation in drug design has become an exciting challenge for medicinal chemistry, as this noncovalent force results in conformational restriction of small drug molecules, leading to increased lipophilicity, membrane permeability and pharmacological activity [8]. Small model systems with intramolecular and/or intermolecular hydrogen bonded amide groups are often employed to study these interactions in solution and in the solid state [9]. Despite the enormous interest in the structural properties of the C-2 *N*-substituted anthranilamide derivatives [10], the presence of intramolecular and/or intermolecular hydrogen bonding in 2-(sulfonylamino)benzamides [11,12] has not been well explored in the literature. This encouraged us to study the structural properties of 2-(benzenesulfonylamino)benzamides in solution and solid state by means of spectroscopic (NMR, UV-Vis and IR) and single crystal X-ray diffraction (XRD) techniques, in combination with the density functional theory (DFT) method in the gas phase. A Hirshfeld surface and a fingerprint plot were generated to explore the intermolecular interactions in the crystalline structure. The intramolecular contacts have also been interpreted using Natural Bond Orbital (NBO) analysis.

## 2. Results and Discussion

### 2.1. Solution Phase Structural Analysis Using NMR and UV-Vis Spectroscopy

**Scheme 1:**
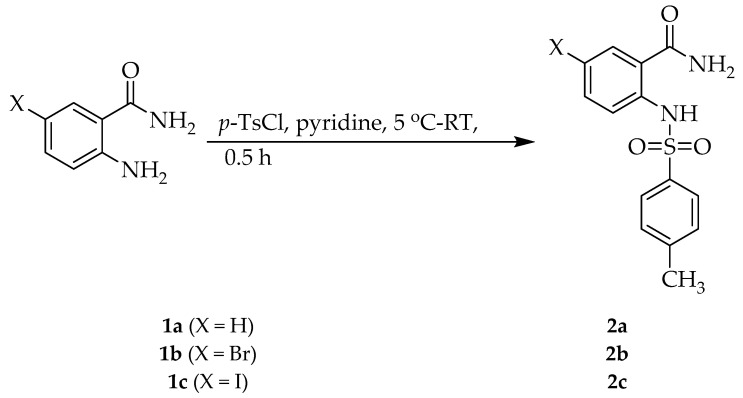
Sulfonylation of anthranilamide derivatives **1a**–**c**.

The test compounds were prepared as shown in Scheme 1 and their structures were determined using a combination of NMR (^1^H- & ^13^C-), UV-Vis, IR and mass spectrometric techniques. The ^1^H- and ^13^C-NMR spectra of compounds **2a**, **2b** and **2c** are enclosed as Figure S1 of the Supplementary Information (SI). The hydrogen atoms of the amide group (–C(O)NH_2_) of the 2-(4-tolylsulfonylamino)benzamides **2a**–**c** resonated as two distinct broad singlets of reduced intensity and different chemical shift values in the aromatic region of their ^1^H-NMR spectra. The observed chemical shift nonequivalence of these amide hydrogen atoms is attributed to the hindered rotation of the H_2_N–C(O) single bond in terms of NMR time scale [13]. A singlet for NH of the arylsulfonamido group resonated significantly downfield around δ = 12.10 ppm due to the deshielding effect of the S=O bonds. This downfield shift of the NH signal is also consistent with the participation of this hydrogen atom in intramolecular hydrogen bond interaction with the carbonyl oxygen.

**Figure 1:**
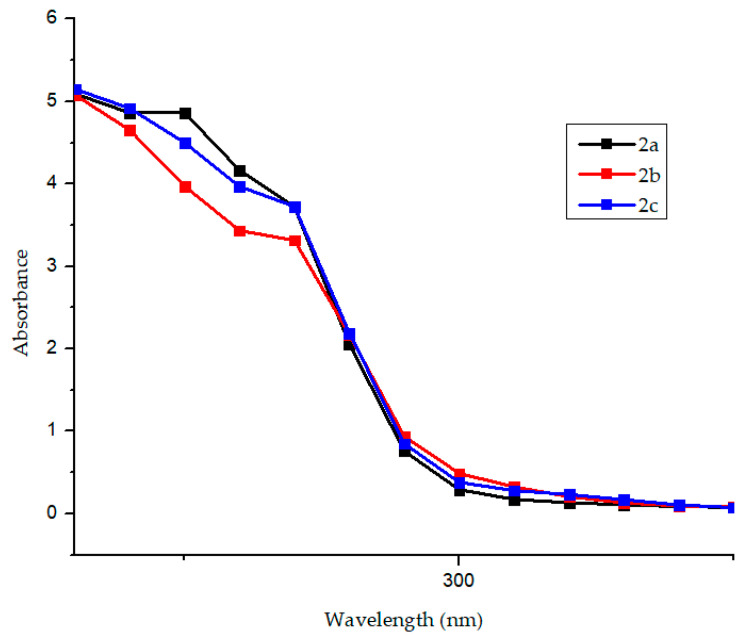
UV-Vis spectra of **2a**, **2b** and **2c** in DMSO (conc. = 1 × 10^−6^ mol/L).

The absorption spectra of the compounds in **2** (Figure 1) were acquired in DMSO at room temperature (200–400 nm) and show bands around around λ = 250 nm and around λ = 275 nm due to π → π* transitions indicative of the presence of benzene chromophore and sulfonamide moiety.

### 2.2. Solid-State Structural Analysis of **3a**–**c** Using IR Spectroscopy and XRD

The FT-IR spectra of the title compounds (refer to Figure S2 in SI) were obtained using the thin-film method, and the corresponding wavenumbers are listed in Table 1 below. The assignments are based on the experimental and the simulated IR spectral data of **2a** as a model (refer to Table S1 for the computed IR frequencies for **2a**–**c**). The computed harmonic vibrational wavenumbers relate to the isolated molecule in a gas phase and are generally greater than the experimentally observed vibrational wavenumbers for molecules in the solid state due to negligence of anharmonicity, electron correlation and basis set deficiencies. The asymmetric and symmetric N–H stretching vibrations of the solid samples of benzenesulfonamide derivatives are observed in the region, ν_max_ = 3444–3473 cm^−1^ and 3345–3371 cm^−1^, respectively, due to intermolecular hydrogen bonding [14]. A band for the N–H symmetric (ν*_sym_* (NH_2_)) stretching vibration of compounds **2** is observed in the region 3300–3400 cm^−1^ of their FT-IR spectra, and these correspond to the N–H stretching mode of the amide group. The corresponding computed symmetric (ν*_sym_* (NH_2_)) stretching vibrations occur at ν_max_ = 3225 cm^−1^ for **2a**, at 3225 cm^−1^ for **2b** and at 3246 cm^−1^ for **2c**. The bands in the region ν_max_ 2921–2971 cm^−1^ are attributed to aromatic and aliphatic C–H stretching vibrations. Strong peaks in the regions of 1313–1385 cm^−1^ and 1149–1161 cm^−1^ are due to the asymmetric and symmetric S=O stretching vibrations, respectively. The computed S=O asymmetric stretching wavenumber occurred at ν_max_ = 1318 and 1382 cm^−1^ for **2a**, at 1321 and 1381 cm^−1^ for **2b**, and at 1318 and 1383 cm^−1^ for **2c**. Although sulfonamides have been found to exhibit S–N stretching vibrations at 924–906 cm^−1^ [14], the corresponding frequencies for **2a**, **2b** and **2c** are ν_max_ = 934 cm^−1^, 939 cm^−1^, and 961 cm^−1^, respectively. The higher stretching vibration values for these compounds presumably reflect the effect of participation of this scaffold in intramolecular hydrogen bonding interaction.

**Figure 2:**
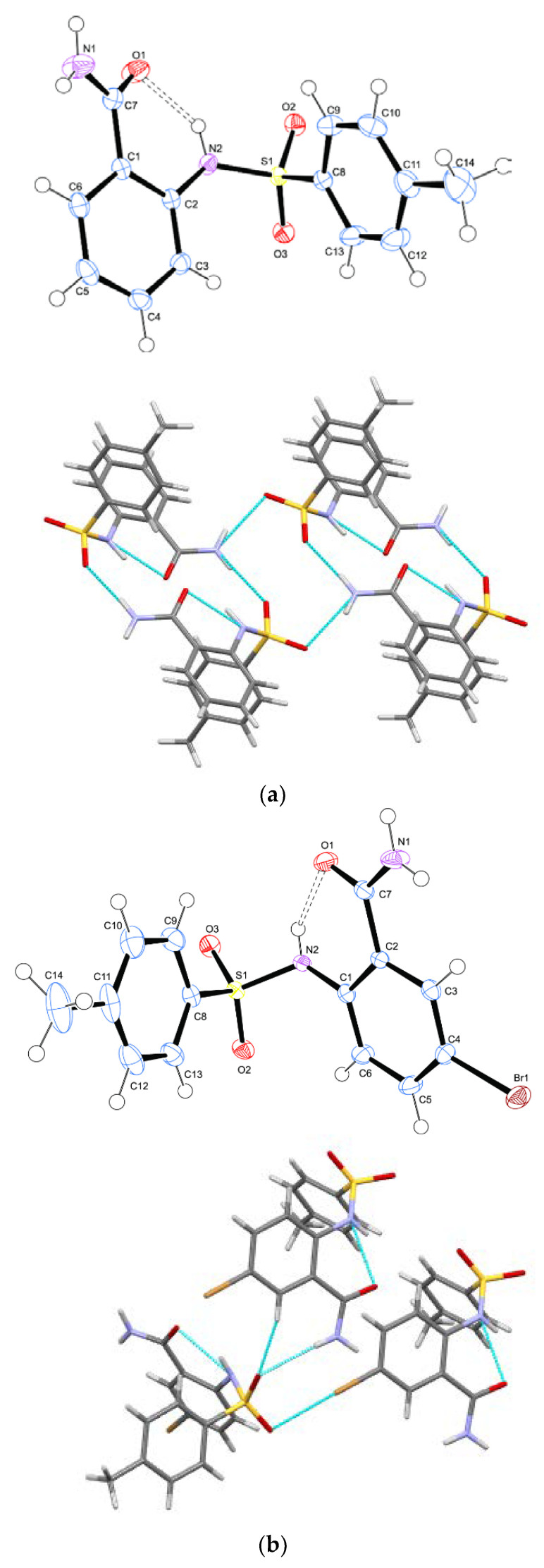

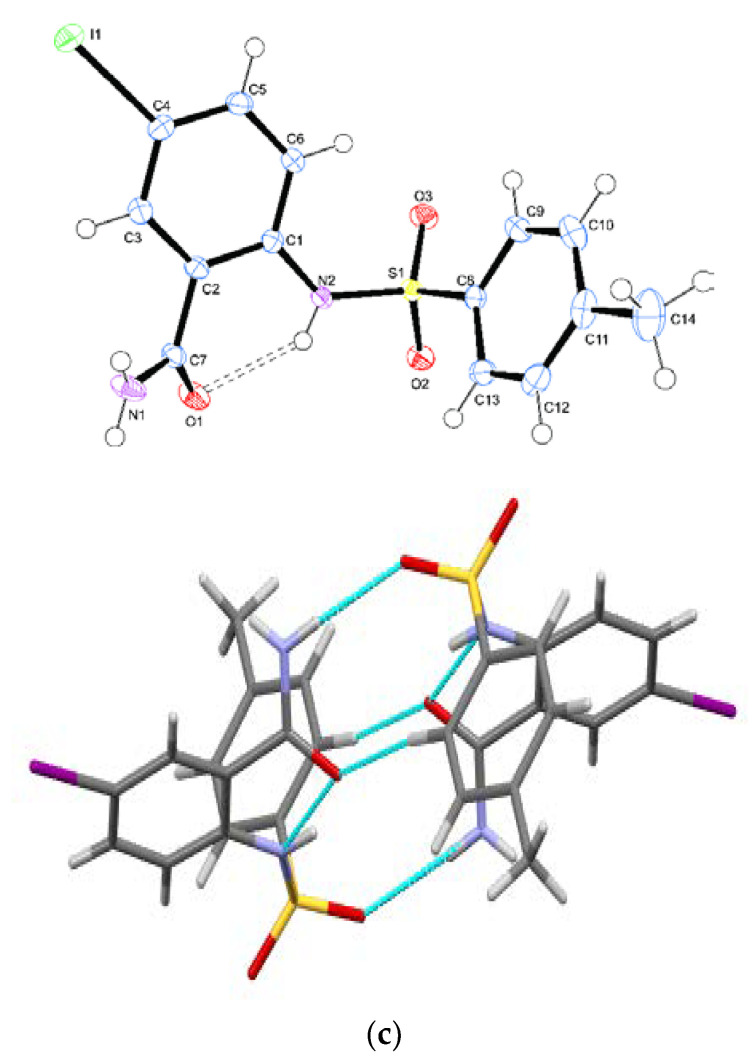
The Oak Ridge Thermal Ellipsoid Plot (ORTEP) diagrams and the X-ray crystal structure of **2a** (**a**), **2b** (**b**) and **2c** (**c**) showing intra- and intermolecular hydrogen bonding.

After confirming the compound homogeneity and purity by NMR and FT-IR spectroscopy, single crystals of compounds **2a**–**c** were subjected to XRD analysis. The crystal structures of compounds **2** are represented in Figure 2 and their atom-labelling schemes differ from systematic numbering. The crystal data and structure refinement of these compounds are presented in Table S2 (refer to SI). Single crystal XRD analysis of **2a**–**c** revealed the presence of intramolecular hydrogen bonding interaction (Table 2) between the oxygen of the amide (–C(O)NH_2_) moiety and the NH of the *p*-tosylsulfonamido group with bond distance N(2)–H(2)^…^O(1) of 1.865(18) Å and bond angle 141(2)° for **2a** (Figure 2a), 1.95(2) and 142(2)° for **2b** (Figure 2b) and 2.09(2) Å and 138(2)° for **2c** (Figure 2c). The hydrogen bonding pattern forms an R^1^_1_(6) motif where R, subscript, superscript and the number in parentheses represent the ring, number of donors, acceptors and number of atoms involved in this hydrogen bonding pattern, respectively. The amide unit of these compounds also functioned as a hydrogen bond donor to form intermolecular hydrogen bonded complexes of the type N–H···O=S, analogous with the literature precedent for anthranilamide derivatives [15]. The propensity of an amide moiety for hydrogen bonding plays an important role in the spatial structure of proteins, nucleic acids and biological membranes, as well as in the interaction of bioactive compounds with receptors [8]. The distorted tetrahedral geometry of the sulfur atom in each case resulted in deviation of the sulfonamide moiety from coplanarity of the anthranilamide scaffold with the following torsion angle between the two rings: C(8)**–**S(1)**–**N(2)**–**C(2) of −53.07(11)° for **2a**, C(1)**–**N(2)**–**S(1)**–**C(8) of 64.18(13)° for **2b** and C(1)**–**N(2)**–**S(1)–C(8) of 75.54(17)° for derivative **2c**. The packing of molecules of compounds **2** occurs via N–H^…^O hydrogen bridges between N(H) atoms of the amide moiety and the oxygen atoms of sulfonyl group of a nearby molecule. This twisted geometry of the sulfonamide group enabled oxygen atoms to form hydrogen bonds in higher dimensions. The conformation of the N(2)–C(2) bond in the C–SO_2_–NH–C segment of the structure of **2a** are *gauche* and *trans* with respect to the S(1)=O(2) and S(1)=O(3) bonds, respectively. Both *anti* and *syn* oxygen atoms of the SO_2_NH group of **2a** are involved in N–H^…^O interactions with the hydrogen atom of the amide (NH_2_CO) moiety of a different molecule to form parallel planes with cyclic motifs R^4^_4_(12) (Figure 2a). The N(2)–C(1) bonds in the C–SO_2_–NH–C segment of the structures of **2b** or **2c**, on the other hand, are *trans* and *gauche* with respect to the S(1)=O(2) and S(1)=O(3) bonds, respectively. The *syn* oxygen atom of the SO_2_NH group of one molecule of **2b** forms a seven-membered cyclic (R^2^_1_(7)) motif with the benzamide scaffold of another molecule of this compound through N–H^…^O=S and C–H^…^O=S bonding interactions (Figure 2b). The C–H^…^O hydrogen bonds play an important role in determining molecular conformation and crystal packing [16], in molecular recognition processes [17], and in the stabilization of inclusion complexes [18]. The *anti* oxygen of this compound is involved in halogen bonding with the bromine atom (O^…^Br) to form a chain. A bromine atom contains a region with positive charge (σ-hole) responsible for its halogen bonding (XB) formation and stabilizing characteristics on the drug molecules [19,20]. Both the *syn* oxygen atom of the SO_2_NH moiety and the oxygen atom of the amide group of one molecule of **2c** are involved in N–H^…^O=S and C=O^…^H–C interactions of the other molecule, respectively, to form a R^2^_2_(8) dimer (Figure 2c). The formation of conventional N–H^…^O bonds seems to be favored over halogen bonding interactions for this compound. It has been proposed that planar molecules show thermochromism, while nonplanar molecules show photochromism [21]. The nonplanar nature of these sulfonamidobenzamides make them suitable candidates for further studies, as solid-state photochromic materials have potential use as photooptical memories and switches.

### 2.3. Computational Studies

#### 2.3.1. Hirshfeld Interactions

**Figure 3:**
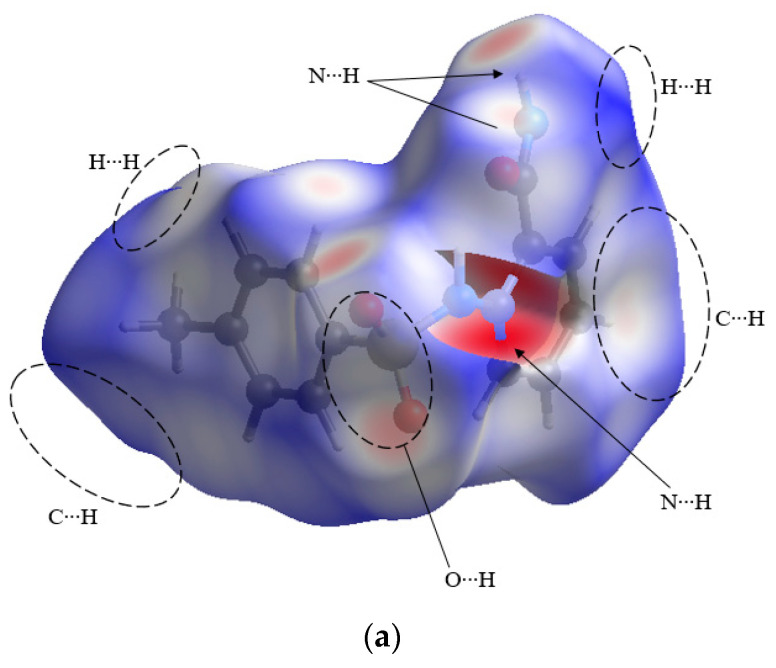

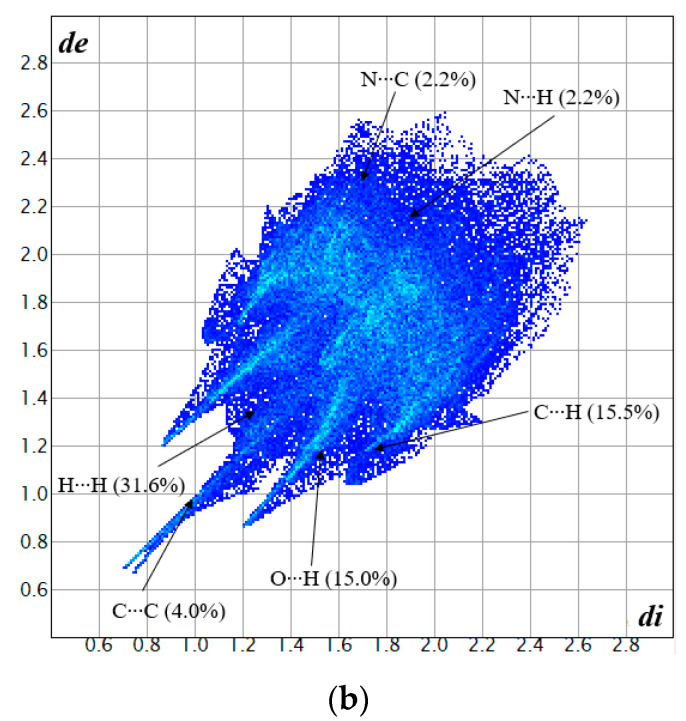
Hirshfeld surface for the crystal structure (**a**) and 2D fingerprint plot analysis (**b**) of **2a**.

Sulfonamides are capable of forming highly ordered supramolecular architectures through hydrogen bonds and π–π stacking interactions [22]. The Hirshfeld surface and 2D fingerprint studies of **2a**–**c** were carried out in order to obtain information about intermolecular contacts and their quantitative contribution to the supramolecular self-assembly. Strong, neutral, and weak interactions in the Hirshfeld surface, which are involved in the stabilization of crystal structure, are depicted as red, white and blue colors, respectively [23]. The Hirshfeld surfaces and two-dimensional (2D) fingerprint plots of these compounds were obtained using the corresponding Common Internet File System’s (CIF’s) files. The Hirshfeld surface and 2D fingerprint plot of **2a** (Figure 3) used as a model (refer to Figure S3 for **2b** and S4 for **2c** in SI) revealed that multiple C–H^…^O=S hydrogen bonds and other hydrogen bonds involving amide and sulfonamide groups played an important role in the assembling processes. Analyses of the Hirshfeld surfaces revealed that the N–H^…^O=C hydrogen bond is the interaction that drives the supramolecular assembly in these compounds, and it is reinforced by H^…^H, O^…^H/H^…^O, C^…^H/H^…^C, C^…^C and N^…^H interactions (Figure 3a). The red spots over the surface indicate the intercontacts involved in hydrogen bonding interactions and interatomic contacts. These intense red spots appeared as sharp spikes in the corresponding fingerprint plots (Figure 3b) and are related to the polar O^…^H hydrogen bonding interactions. The faded red spots in the d_norm_ map, and the broad peaks in the fingerprint plots are due to the hydrophobic C^…^H and H^…^H interactions. The percentages of the contacts that contribute to the total Hirshfeld surface of **2a** are as follows: H^…^H (31.6%), O^…^H/H^…^O (15.0%), C^…^H/H^…^C (15.5%), C^…^C (4.0%), N^…^H (2.2%). The major contributions of the total area of the surface are from H^…^H (31.6%), C^…^H/H^…^C (15.5%) and O^…^H/H^…^O (15.0%) interactions compared to the other intercontacts. The contribution percentages to the total Hirshfeld surface of **2b** (refer to Figure S4 in SI) listed by contact are H^…^H (31.4%), O^…^H/H^…^O (11.5%), C^…^H (10.2%) and Br^…^H (8.6%), C^…^O (0.9%), Br^…^O/O^…^Br (2.2%) and N^…^H (1.8%). Those for **2c** (refer to Figure S4 in SI) are as follows: H^…^H (36.3%), O^…^H/H^…^O (9.5%), C^…^H (8.3%) and I^…^H (7.8%), C^…^O (4.6%) and I^…^I (2.6%). Although there is no halogen bonding interaction for this compound, iodine atoms are engaged in halogen–halogen interaction.

#### 2.3.2. Optimized Structures of **2a**, **2b** and **2c** from DFT Analyses

**Figure 4:**
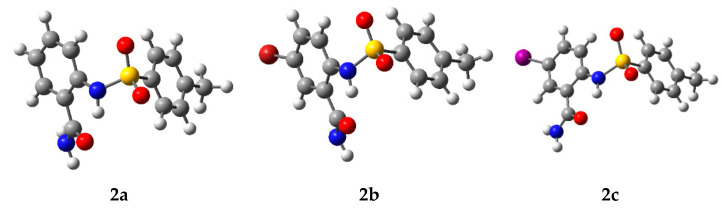
Optimized structures of **2a**, **2b** and **2c** in the gas phase.

The optimized geometries were obtained in the gas phase using a B3LYP/6-311G (d,p) basis set for **2a** and **2b**, and a B3LYP/LanL2DZ basis set for **2c**. The minimum potential energy was achieved by solving the self-consisting field interactively (local minimum) to obtain the corresponding geometries shown in Figure 4. The theoretically calculated values of the bond angles, dihedral angles and torsion angles of the optimized structures of **2a**, **2b** and **2c** are given in Table S3 in the Supplementary Information. Selected experimental and calculated bond lengths, bond angles and torsion angles of compound **2a** (used as a representative model) are represented in Table 3 below. The results revealed a good agreement between the calculated geometric parameters (bond distances and bond angles) of compounds **2a**, **2b** and **2c** with those obtained from the CIFs. The differences observed between the experimental (XRD) and calculated (DFT) parameters is due to the gas phase and theoretical methods ignoring molecular interactions observed in the solid state. A short C(7)–N(1) bond length of about 1.33 Å for the test compounds (which is significantly shorter than the value (1.470 Å) proposed for the C(sp^2^)–N(sp^2^) single bond length) [24] may be indicative of a double bond character. This, in our view, may be the cause of the hindered rotation about the C(sp^2^)–N(sp^2^) single bond and, therefore, the nonequivalence of the resonances for the amide hydrogen atoms observed in the ^1^H-NMR spectra of these compounds. However, a value of about 1.322 Å observed for the C–N bonds in some amides has previously also been suggested to be indicative of the single bonded C(sp^2^)–N(sp^2^) [25].

**Figure 5:**
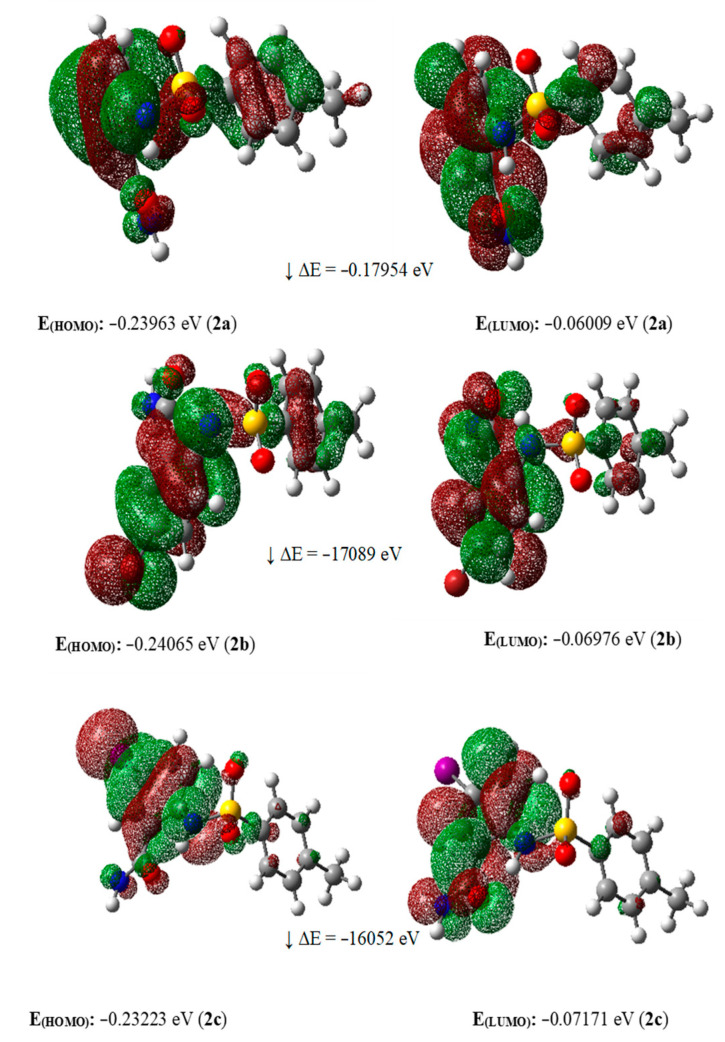
HOMO–LUMO distribution of **2a**, **2b** and **2c**, and the energy gap.

The Highest Occupied Molecular Orbital (HOMO) and Lowest Unoccupied Molecular Orbital (LUMO) surfaces of the most stable conformers of **2a**, **2b** and **2c** are illustrated in Figure 5. DFT calculations revealed that the HOMO is mainly localized on the anthranilamide scaffold and significantly or partially localized on the *p*-toluenesulfonamide arm for **2a** (Figure 5) and **2b** (Figure 5), respectively. The HOMO for **2c** (Figure 5) is largely distributed throughout the sulfonamidobenzamide framework and not on the *p*-tolyl group. The LUMO of the three compounds is localized mainly on the sulfonamidobenzamide framework. The energy gap between the HOMO and LUMO displays the delocalization of the π-electron cloud from the donor to the acceptor unit, respectively. The electronic transition occurred between the frontier molecular orbitals from HOMO to LUMO, and the small computed energy gap (ΔE) values for **2a** (−0.17954 eV), **2b** (−0.17089 eV) and **2c** (−0.16052 eV) show that these compounds are soft molecules that can be excited using minimal energy. The strong charge transfer interaction through the π-conjugated system led to substantial ground state donor–acceptor mixing and the appearance of a charge transfer band in the electron absorption spectrum.

A partial double bond character for the C–N bond of primary amides is always invoked to account for the hindered rotation [24], and is therefore the cause of the nonequivalence of the resonances for the amide hydrogen atoms observed in their ^1^H-NMR spectra [13,15]. Although the resonance interaction model provides a convenient explanation for the stabilization of the amino–carbonyl moiety, the changes in electron population and structure on rotation were previously found not to be in accord with an electron donation from the amino group to the carbonyl oxygen of formamide [26]. Consequently, the natural bond orbitals (NBOs) analysis of compounds **2a**, **2b** and **2c** were performed at the B3LYP/6-311G (d,p) level of theory (**2a** and **2b**) and at the B3LYP/ LanL2DZ (d,p) level of theory (**2c**) to further elucidate the delocalization of electron density within these molecules (Table S4 in SI). The interactions between the bonding n1(O_3_) and antibonding (N_2_–S_1_) orbitals of compounds **2a**, **2b** and **2c** show the delocalization of charge in the sulfonamide framework with stabilization energy values of 1.43, 1.81 and 2.28 kJ.mol^−1^, respectively. These compounds exhibit intramolecular hyperconjugation formed by an overlap between n1 (N_1_) σ bonding and π* (C_7_–O_1_) antibonding orbitals, which is stabilized by the intramolecular charge transfer (ICT) with stabilization energies of 58.1 (**2a**), 51.4 (**2b**) and 61.9 kJ.mol^−1^ (**2c**). There is an increase in electron density (ED) into the C–O and C–N antibonding orbitals, which elongates the nitrogen–carbon bond. The changes in bond lengths in C=O and C–N are consistent with the decrease in the double bond character of C=O bond due to hydrogen bonds, which, in turn, increase the double bond character of C–N bond [27].

## 3. Materials and Methods

### 3.1. Chemical Synthesis

#### 3.1.1. Preparation of Substrates

Anthranilamide (**1a**) was purchased from Sigma-Aldrich (Sigma-Aldrich, Bradford, UK). 2-Amino-5-bromoanthranilamide (**1b**) [28] and 2-amino-5-iodoanthranilamide (**1b**) [29] were prepared following methods described in our previous studies. The most convenient and efficient method for the synthesis of sulfonamides involves the use of alkyl/arylsulfonyl chlorides and amines as starting materials at room or elevated temperature in the presence of appropriate solvent and/or amine bases to scavenge the HCl generated from the reaction [2]. The outcome of this reaction depends on the reaction temperature, time, and quantities of the sulfonyl chloride and amine base used. High temperature and prolonged reaction time facilitated the attachment of a second sulfonyl chloride reagent on the aniline nitrogen atom and/or dehydration of the amide NH_2_ group into a nitrile group [12,30]. The 2-(*p*-tolylsulfonamido)benzamides **2a**–**c** were prepared as described below.

#### 3.1.2. Typical Procedure for the Synthesis of the 2-(*p*-Tolylsulfonamido)benzamides **2a**–**c**

*p*-Toluenesulfonyl chloride (1.2 equiv.) was added gradually to a stirred solution of anthranilamide derivative **1** (1 equiv.) in pyridine (2 mL/mmol of **1**) at 0–5 °C. The mixture was allowed to warm to room temperature and, after 0.5 h at this temperature, the mixture was quenched with an ice-cold water. The precipitate was filtered and recrystallized from acetonitrile to afford a pure solid product.

*2-((4-Methylphenyl)sulfonamido)benzamide* (**2a**), White solid (1.65 g, 92%), mp. 227–230 °C (CH_3_CN) (Lit. [12] 250–251 °C); ^1^H-NMR (DMSO-*d*_6_) 2.30 (3H, s, −CH_3_), 7.03 (1H, t, *J* = 7.5 Hz, H-5), 7.29 (2H, dd, *J* = 2.0 and 8.0 Hz, H-3’,5’), 7.40 (1H, t, *J* = 8.0 Hz, H-4), 7.49 (1H, d, *J =* 8.5 Hz, H-3), 7.62 (2H, d, *J* = 8.5 Hz, H-2’,6’), 7.74 (1H, d, *J* = 8.0 Hz, H-6), 7.78 (1H, br s, −NHCO), 8.27 (1H, br s, –NHCO), 12.16 (1H, s, −NHS); ^13^C-NMR (DMSO-*d*_6_) 21.4, 119.2, 119.4, 123.2, 123.3, 127.2, 129.4, 130.1, 130.2, 133.1, 133.2, 136.6, 139.8, 144.0, 171.1; HRMS (ES): *m*/*z* [M + H]^+^ calc 291.0725 for C_14_H_15_N_2_O_3_S; found 291.0759.

*5-Bromo-2-((4-methylphenyl)sulfonamido)benzamide* (**2b**), White solid (1.72 g, 96%), mp. 194–196 °C (CH_3_CN); ^1^H-NMR (DMSO-*d*_6_) 2.29 (3H, s, −CH_3_), 7.33 (2H, d, *J* = 7.0 Hz, H-3’,5’), 7.46 (1H, d, *J* = 8.0 Hz, H-3), 7.63 (3H, d, *J* = 8.5 Hz, H-4 and H-2’,6’), 7.97 (1H, d, *J* = 1.5 Hz, H-6), 7.99 (1H, br s, –NHCO), 8.41 (1H, br s, −NHCO), 12.06 (1H, s, −NHS); ^13^C-NMR (DMSO-*d*_6_) 21.4, 119.6. 121.4, 121.5, 127.2, 130.4, 131.9, 135.5, 136.1, 138.9, 144.5, 169.7; HRMS (ES): *m*/*z* [M + H]^+^ calc 368.9830 for C_14_H_14_BrN_2_O_3_S; found 368.9810.

*5-Iodo-2-((4-methylphenyl)sulfonamido)benzamide* (**2c**)*,* White solid (1.45 g, 89%), mp. 186–188 °C (CH_3_CN); ^1^H-NMR (DMSO-*d*_6_) 2.30 (3H, s, −CH_3_), 7.29 (2H, d, *J* = 8.5 Hz, H-3’,5’), 7.30 (1H, d, *J* = 9.0 Hz, H-3), 7.61 (2H, d, *J* = 8.5 Hz, H-2’,6’), 7.71 (1H, dd, *J* = 1.5 and 9.0 Hz, H-4), 7.87 (1H, br s, −NHCO), 8.07 (1H, d, *J* = 1.5 Hz, H-6), 8.35 (1H, br s, −NHCO), 12.04 (1H, s, −NHS); ^13^C-NMR (DMSO-*d*_6_) 21.5, 87.1, 121.3, 121.4, 127.2, 130.2, 136.3, 137.4, 139.4, 141.5, 144.2, 169.7; HRMS (ES): *m*/*z* [M + H]^+^ calc 416.9692 for C_14_H_14_IN_2_O_3_S; found 416.9225.

### 3.2. Instrumentation

The ^1^H- and ^13^C-NMR spectra of the title compounds were obtained as DMSO-*d*_6_ solutions using an Agilent 500 MHz NMR spectrometer (Agilent Technologies, Oxford, UK) and the chemical shifts are quoted relative to the tetramethylsilane (TMS) peak. The UV-vis spectra were recorded on a Cecil CE 9500 (9000 Series) UV-Vis spectrometer (ThermoFisher Scientific, Waltham, MA, USA). The IR spectra of the test compounds were recorded at room temperature by using the thin-film method on a Bruker VERTEX 70 FT-IR Spectrometer (Bruker Optics, Billerica, MA, USA) equipped with a diamond attenuated total reflectance (ATR) accessory. The high-resolution mass spectra were recorded at an ionization potential of 70 eV using a Micromass Autospec-TOF (double focusing high resolution) instrument (Waters Corp., Milford, MA, USA).

### 3.3. Data Collection and Refinement

Intensity data were determined on a Bruker Venture D8 Photon CMOS diffractometer with graphite-monochromated MoKa_1_ (l = 0.71073 Å) radiation at 173 K using an Oxford Cryostream 600 cooler. Data reduction was carried out using the program SAINT+, version 6.02 [31] and empirical absorption corrections were made using SADABS [31]. Space group assignments was made using XPREP [31]. The structure was solved in the WinGX [32] suite of programs, using intrinsic phasing through SHELXT [32] and refined using full-matrix least-squares/difference Fourier techniques on F^2^ using SHELXL-2017 [33]. All C bound hydrogen atoms were placed at idealized positions and refined as riding atoms with isotropic parameters 1.2 times those of their parent atoms. O– and N–bound hydrogen atoms were located in the difference Fourier map and their coordinates and isotropic displacement parameters refined freely. Diagrams and publication material were generated using ORTEP-3 [32], and PLATON [34].

### 3.4. Computational Methods

The structures were fully optimized by density functional theory using the Gaussian 09 program package (Gaussian Inc., revision D.01, Wallingford, CT, USA) [35] in the gas phase. The DFT computations were carried out using the hybrid functional Becke’s three-parameter nonlocal exchange functional with the Lee–Yang–Parr correlation function (B3LYP) [36], together with the 6-311++G(d,p) [37,38] basis set for all atoms and the LanL2DZ ECP basis set for the iodine atom [39,40].

### 3.5. Hirshfeld Surface Analyses

The three-dimensional Hirshfeld (HF) surfaces and two-dimensional (2D) fingerprint plots of the title compounds were carried out using the Crystal Explorer software [41]. The normalized contact distance (d_norm_) is based on the distance from the point to the nearest nucleus external to the surface (d_e_) and the distance to the nearest nucleus internal to the surface (d_i_) [42].

## 4. Conclusions

The results of this study demonstrate that the conformations of 2-((4-methylphenyl)sulfonamido)benzamides in solution and in the solid state are controlled by inter- and intramolecular networks of different types of hydrogen bonding. ^1^H-NMR spectroscopy showed that the amide protons resonate in different chemical shift environments, and their nonequivalence is due to hindered rotation of the C(O)–NH_2_ single bond. Significant downfield shift of the resonance for the sulfonamide hydrogen in the ^1^H-NMR spectra of these compounds is due to its involvement in intramolecular hydrogen bonding interaction with the carbonyl oxygen and its proximity to the strongly electron withdrawing -SO_2_- group. Use of the XRD method provided unambiguous proof of the existence of a six-membered intramolecular hydrogen bonded ring (graph set descriptor, R^2^_2_(6)) involving hydrogen atom of the sulfonamide group and carbonyl oxygen. Moreover, XRD analyses confirmed the ability of the amide moiety of these compounds to function as a hydrogen bond donor and acceptor simultaneously. The distorted tetrahedral geometry of the sulfur atom caused the sulfonamide group to be twisted out of coplanarity of the intramolecularly hydrogen bonded anthranilamide scaffold. This twisted geometry enabled the oxygen atoms of the sulfonamide group to form hydrogen bonds in higher dimensions than the amide group. Despite conformational similarities, the molecular packing schemes of the test compounds have been found to differ considerably depending on the intermolecular interactions present in their crystal lattices. The capability of the sulfonamidobenzamide scaffold to engage in noncovalent and hydrophobic interactions make the title compounds suitable candidates for deployment in medicinal chemistry. The small HOMO–LUMO energy gap of these compounds, on the other hand, make them attractive targets for future studies of nonlinear optical (NLO) properties. Likewise, the nonplanar nature of this scaffold make these compounds suitable candidates for further studies as solid-state photochromic materials with potential applications as photooptical memories and switches.

## Figures and Tables

**Table 1:** The experimental IR frequencies (cm^−1^) for compounds **2a**–**c**.

2a–c	N–H asym/sym str	C–H	C=O	N–H bend	S=O asym/sym str	S–N
**2a**	3469/3362	2971	1652	1493	1313/1153	935
**2b**	3473/3371	2921	1662	1485	1327/1161	939
**2c**	3444/3345	2931	1668	1498	1318/1149	961

**Table 2:** Hydrogen bond geometries (Å and °) of compounds **2a**, **2b** and **2c**.

D–H^…^A	d(D–H)	d(H^…^A)	d(D^…^A)	<(DHA)
**2a**				
N(1)–H(1A)^…^O(2)#1	0.886(19)	2.180(19)	3.0405(15)	164(2)
N(1)–H(1B)^…^O(3)#2	0.86(2)	2.23(2)	3.0542(15)	161(2)
N(2)–H(2)^…^O(1)	0.873(18)	1.865(18)	2.6045(14)	141(2)
**2b**				
N(1)–H(1A)…O(2)#1	0.87(2)	2.17(2)	2.9690(18)	151(2)
N(1)–H(1B)…O(3)#2	0.83(2)	2.33(2)	3.1462(19)	170(2)
N(2)–H(2)…O(1)	0.77(2)	1.95(2)	2.6022(17)	142(2)
**2c**				
N(1)–H(1A)^…^O(2)#1	0.82(3)	2.14(3)	2.947(2)	172(2)
N(1)–H(1B)^…^O(1)#2	0.77(2)	2.23(3)	2.973(2)	164(2)
N(2)–H(2)^…^O(1)	0.80(2)	2.09(2)	2.7359(19)	138(2)

**Table 3:** Selected XRD and density functional theory (DFT) bond lengths (Å), bond angles (°) and dihedral angles (°) for **2a**.

	Experimental	DFT
Bond Lengths (Å)		
**2a:** C(7)=O(1), N(2)–S(1)	1.2410 (15), 1.6224 (10)	1.230, 1.689
O(2)–S(1), O(3)–S(1)	1.4399 (9), 1.4381 (9)	1.458, 1.455
C(7)–N(1), C(8)–S(1)	1.3331 (16), 1.7555 (12)	1.367, 1.798
**2b:** C(7)=O(1), N(2)–S(1)	1.2381 (18), 1.6318 (12)	1.229, 1.692
O(2)–S(1), O(3)–S(1)	1.4344 (11), 1.4363 (11)	1.454, 1.458
C(7)–N(2), C(8)–S(1)	1.333 (2), 1.7585 (15)	1.365, 1.797
**2c:** C(7)=O(1), N(2)–S(1)	1.242 (2), 1.6306 (14)	1.274, 1.624
O(2)–S(1), O(3)–S(1)	1.6306(14), 1.4299(12)	1.628, 1.628
C(7)–N(2), S(1)–C(8)	1.328 (2), 1.7574 (17)	1.374, 1.784
**Bond Angles (°)**		
**2a:** N(2)–S(1)–C(8)	107.84 (5)	107.50
O(2)–S(1)–C(8)	109.43 (5)	108.20
O(3)–S(1)–C(8)	107.47 (5)	105.59
C(2)–N(2)–S(1)	126.06 (8)	125.58
**2b:** N(2)–S(1)–C(8)	107.51 (7)	107.51
O(2)–S(1)–C(8)	107.71 (7)	107.66
O(3)–S(1)–C(8)	108.45 (7)	108.35
C(1)–N(2)–S(1)	123.74 (10)	123.74
**2c:** N(2)–S(1)–C(8)	107.08 (8)	107.20
O(2)–S(1)–C(8)	108.51 (8)	108.47
O(3)–S(1)–C(8)	107.49 (8)	107.28
C(1)–N(2)–S(1)	130.22 (12)	129.81
**Torsion Angles (°)**		
**2a:** C(8)–S(1)–N(2)–C(2)	–53.07 (11)°	−57.10
C(3)–C(2)–N(2)–S(1)	−32.18 (16)	−32.38
C(9)–C(8)–S(1)–N(2)	−66.54 (11)	−66.05
O(2)–S(1)–N(2)–C(2)	−169.35 (10)	−165.75
**2b:** C(1)–N(2)–S(1)–C(8)	64.18 (13)°	64.54
C(6)–C(1)–N(2)–S(1)	52.08 (18)	52.26
C(9)–C(8–S(1)–N(2)	66.41 (14)	66.14
C(1)–N(2)–S(1)–O(3)	179.24 (12)	179.70
**2c:** C(1)–N(2)–S(1)–C(8)	75.54 (17)°	75.54
C(6)–C(1)–N(2)–S(1)	11.00 (3)	12.50
C(9)–C(8)–S(1)–N(2)	−110.21 (15)	110.73
C(1)–N(2)–S(1)–O(3)	−40.99 (18)	−40.99

## Data Availability

No other data were reported.

## Supplementary Materials

Copies of the ^1^H-NMR and ^13^C-NMR spectra (Figure S1), IR spectra (Figure S2), computed IR spectral data (Table S1), crystal data and structure refinement for compounds **2a**–**c** (Table S2), the Hirshfeld surface and 2D fingerprint plot (Figures S3 and S4), the calculated geometric parameters (bond lengths and angles) of **2a**–**c** (Table S3), and second-order perturbation theory analysis of the Fock matrix in NBO basis for compounds **2a**–**c** (Table S4) The CIF files containing complete information on the studied structures were deposited with the Cambridge Crystallographic Data Center, CCDC 2043901 (**2a**), CCDC 2049537 (**2b**) and CCDC 2049356 (**2c**), and are freely available upon request from the following website: www.ccdc.cam.ac.uk/datarequest/cif or by contacting the Cambridge Crystallographic Data Centre, 12, Union Road, Cambridge CB2 1EZ, UK; fax: +44-1223-336033; email: deposit@ccdc.cam.ac.uk.
