# Supplementary material for: Spectroscopic, X-ray Diffraction and Density Functional Theory Study of Intra- and Intermolecular Hydrogen Bonds in Ortho-(4-tolylsulfonamido)benzamides
Source: Molecules. 2021 Feb 10;26(4):926. doi: 10.3390/molecules26040926 (PMC7916388; doi:10.3390/molecules26040926)
Supplement: Supplementary file 1 [file molecules-26-00926-s001.pdf]

Supplementary Information

Figure S1: ^{1}H- and ^{13}C-NMR spectra of **2a**, **2b** and **2c.**

Figure S2: FT-IR spectra of **2a**, **2b** and **2c.**

Table S1: Computed IR spectral data of **2a**, **2b** and **2c.**

Table S2: Crystal data and structure refinement for compounds **2a–c**.

Figure S3: The Hirshfeld surface and 2D fingerprint plot of **2b** and **2c.**

Table S3: Calculated bond lengths and bond angles of **2a, 2b** and **2c.**

Table S4: Second-order perturbation theory analysis of Fock matrix in NBO basis for **2a–c.**

Figure S1: ^{1}H- and ^{13}C-NMR spectra of **2a**, **2b** and **2c.**

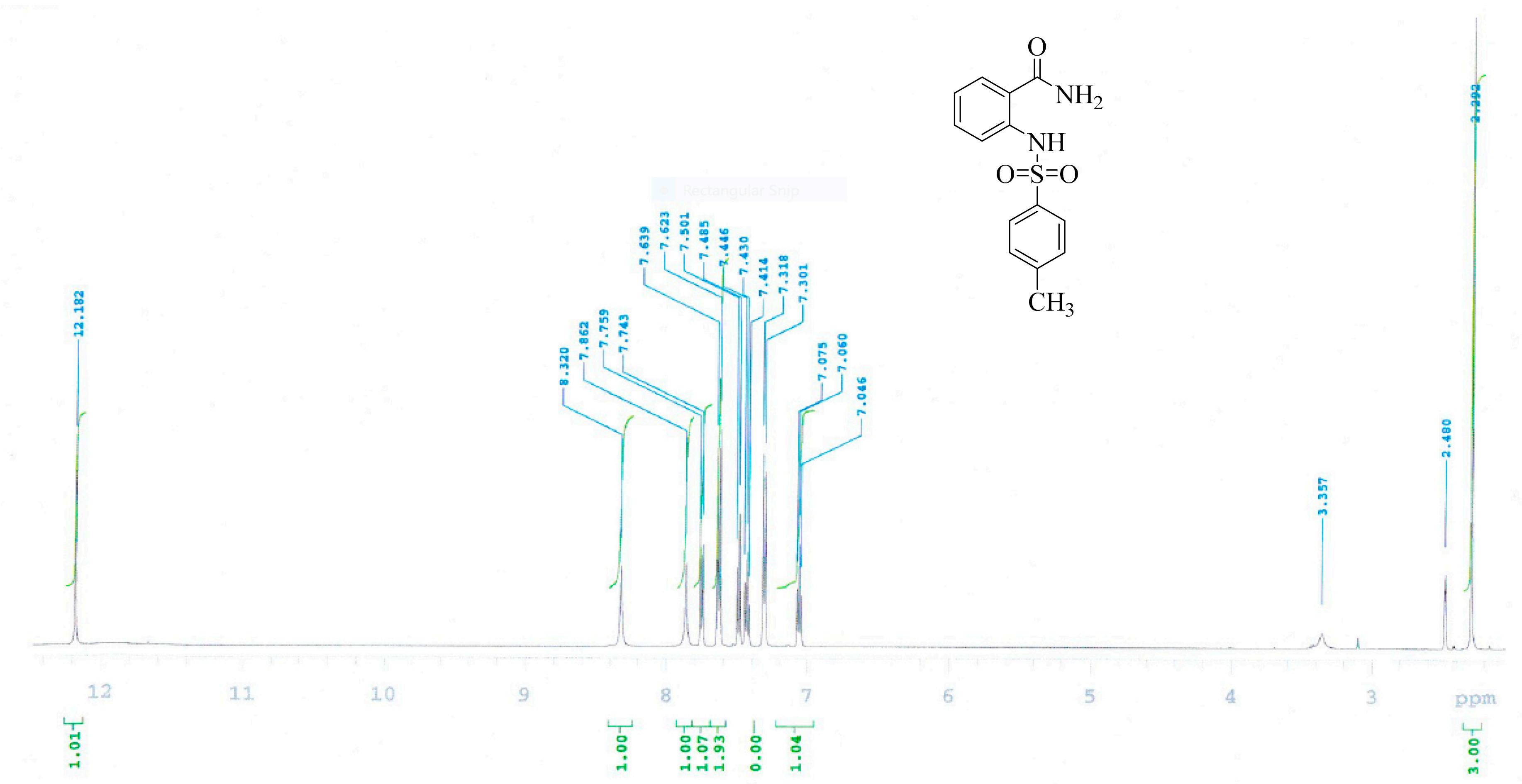

(a)

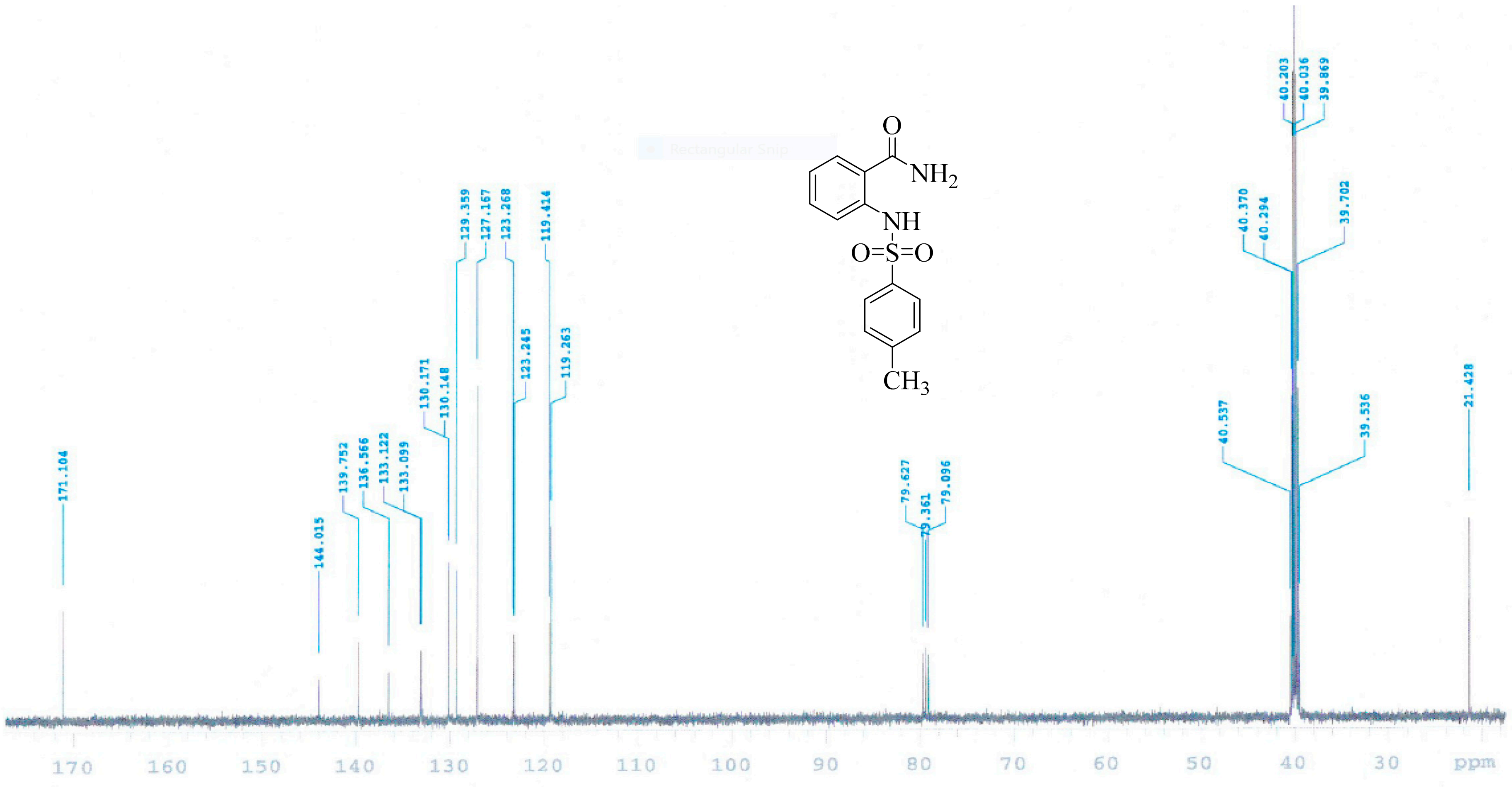

(b)

Figure S1.1: ^{1}H- and ^{13}C-NMR spectra of **2a** in DMSO-d_6 at 500 MHz and 125 MHz, respectively.

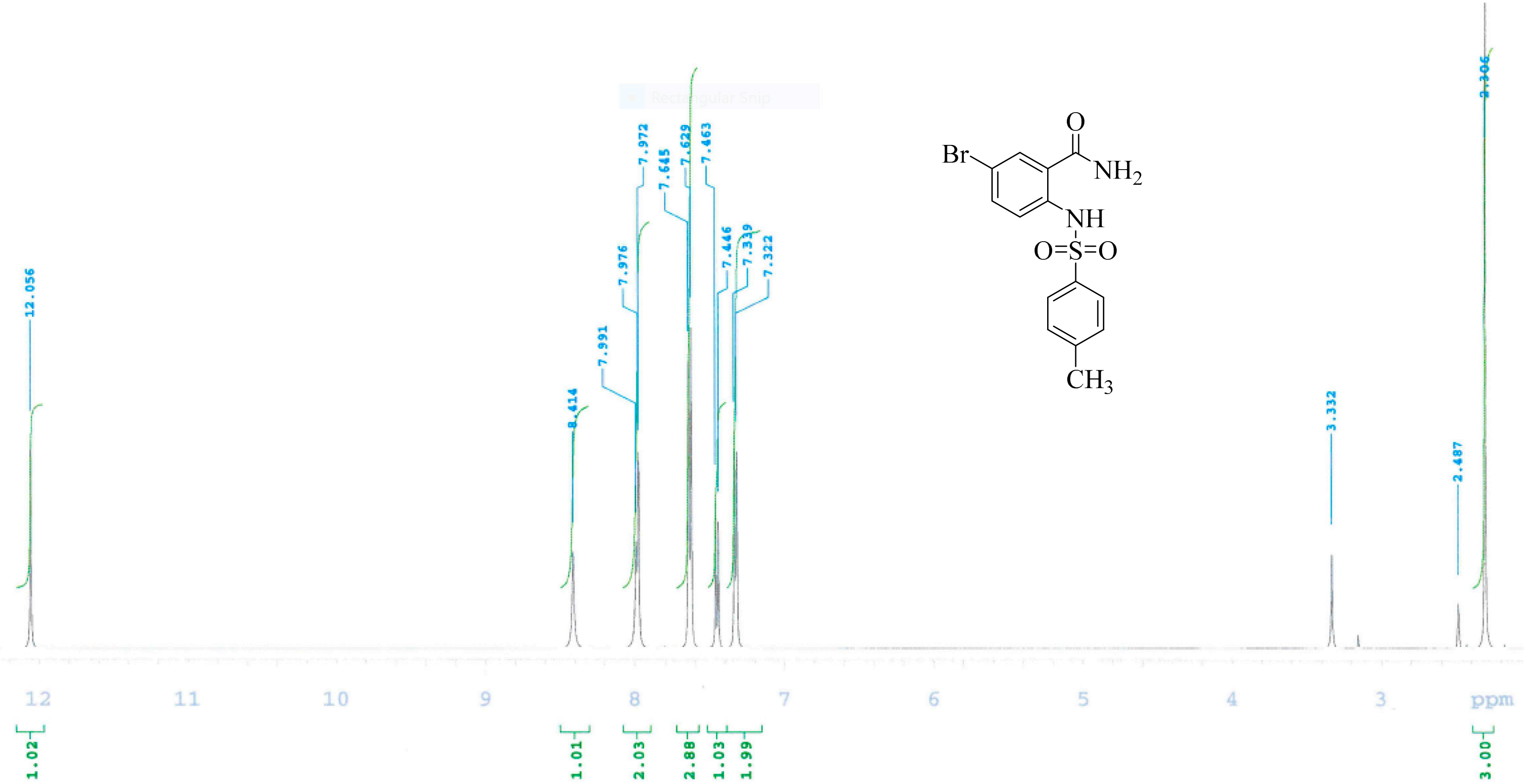
12.056
8.414
7.991
7.976
7.972
7.645
7.629
7.463
7.446
7.339
7.322
3.332
2.487
2.306
12
11
10
9
8
7
6
5
4
3
ppm
1.02
1.01
2.03
2.88
1.03
1.99
3.00
Br
O
NH2
NH
O=S=O
CH3

(a)

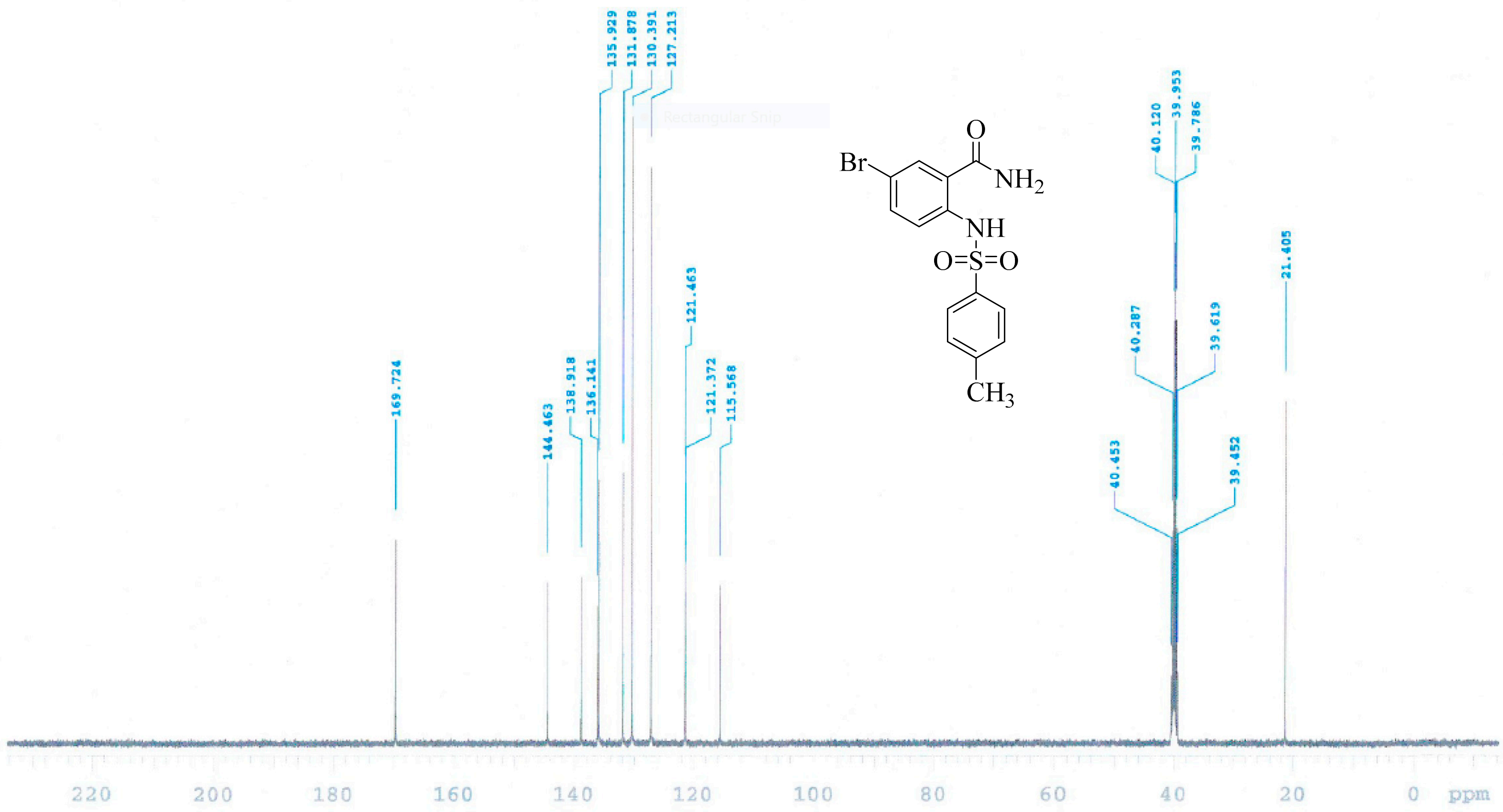

(b)

Figure S1.2: ^{1}H- and ^{13}C-NMR spectra of **2b** in DMSO-d_6 at 500 MHz (a) and 125 MHz (b), respectively.

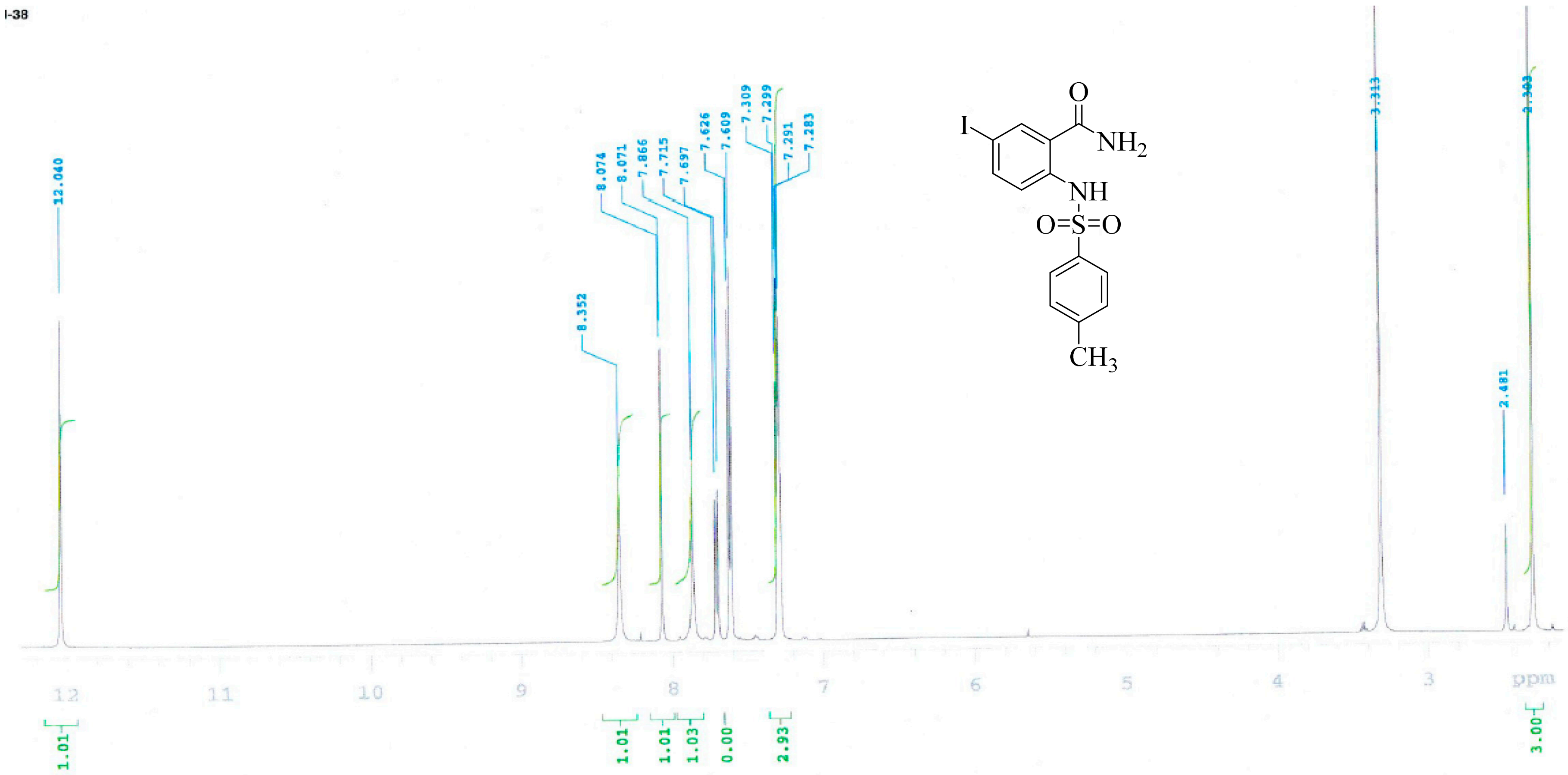
I-38
O
I
NH2
NH
O=S=O
CH3
12.040
8.352
8.074
8.071
7.866
7.715
7.697
7.626
7.609
7.309
7.299
7.291
7.283
3.313
2.481
2.303
12
11
10
9
8
7
6
5
4
3
ppm
1.01
1.01
1.01
1.03
0.00
2.93
3.00

(a)

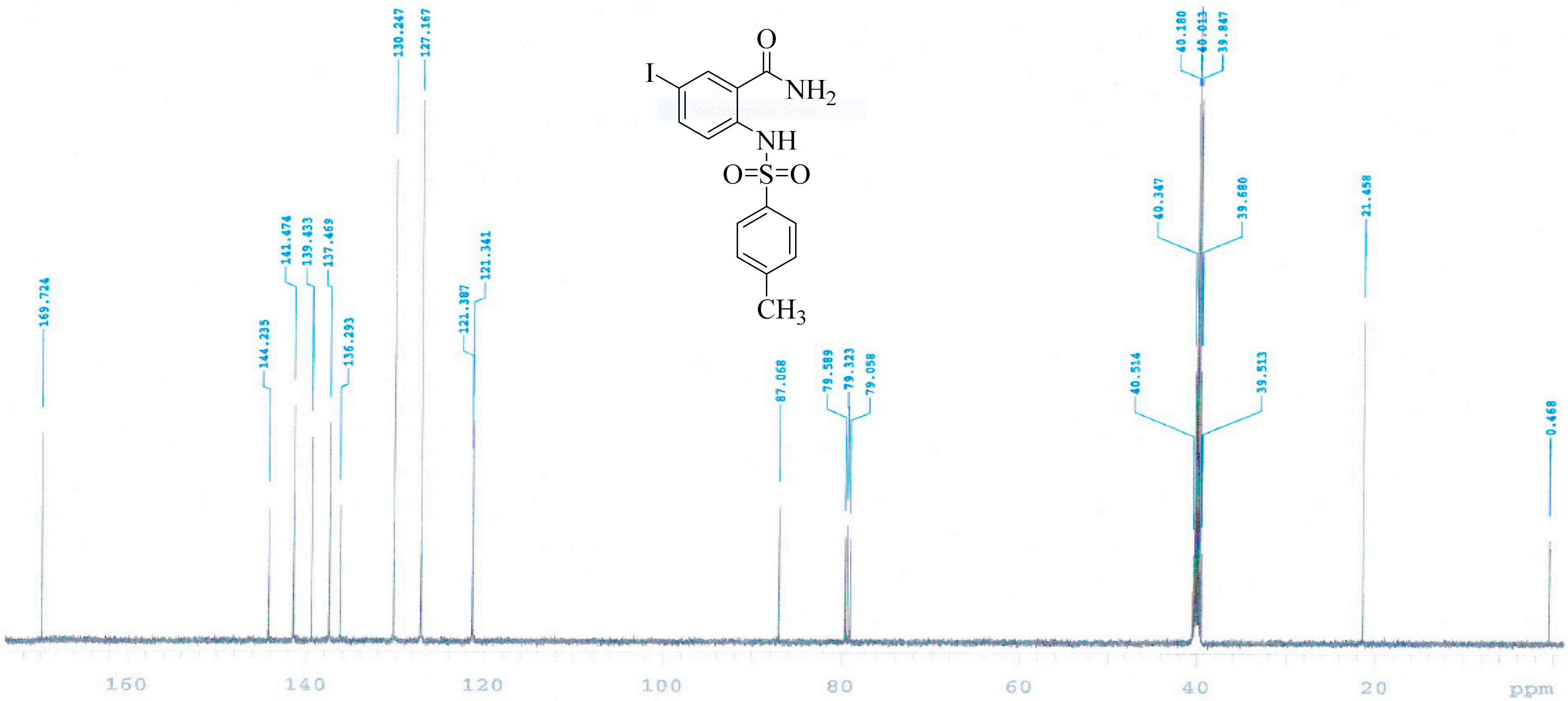

(b)

Figure S1.3: ^{1}H- and ^{13}C-NMR spectra of **2c** in DMSO-d_6 at 500 MHz (a) and 125 MHz (b), respectively.

Figure S2: FT-IR spectra of **2a**, **2b** and **2c**.

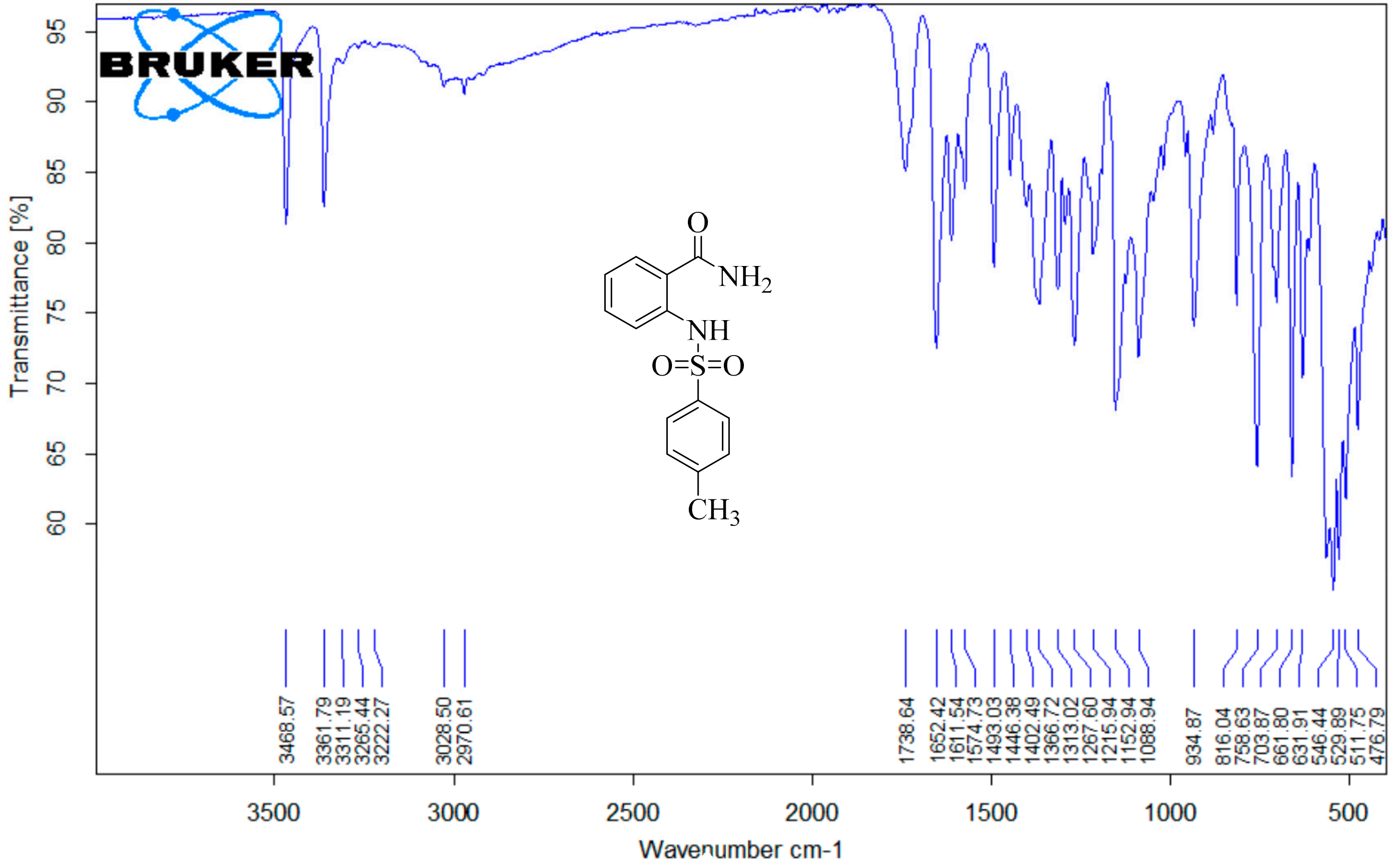

Figure S2.1: FT-IR spectrum of **2a**.

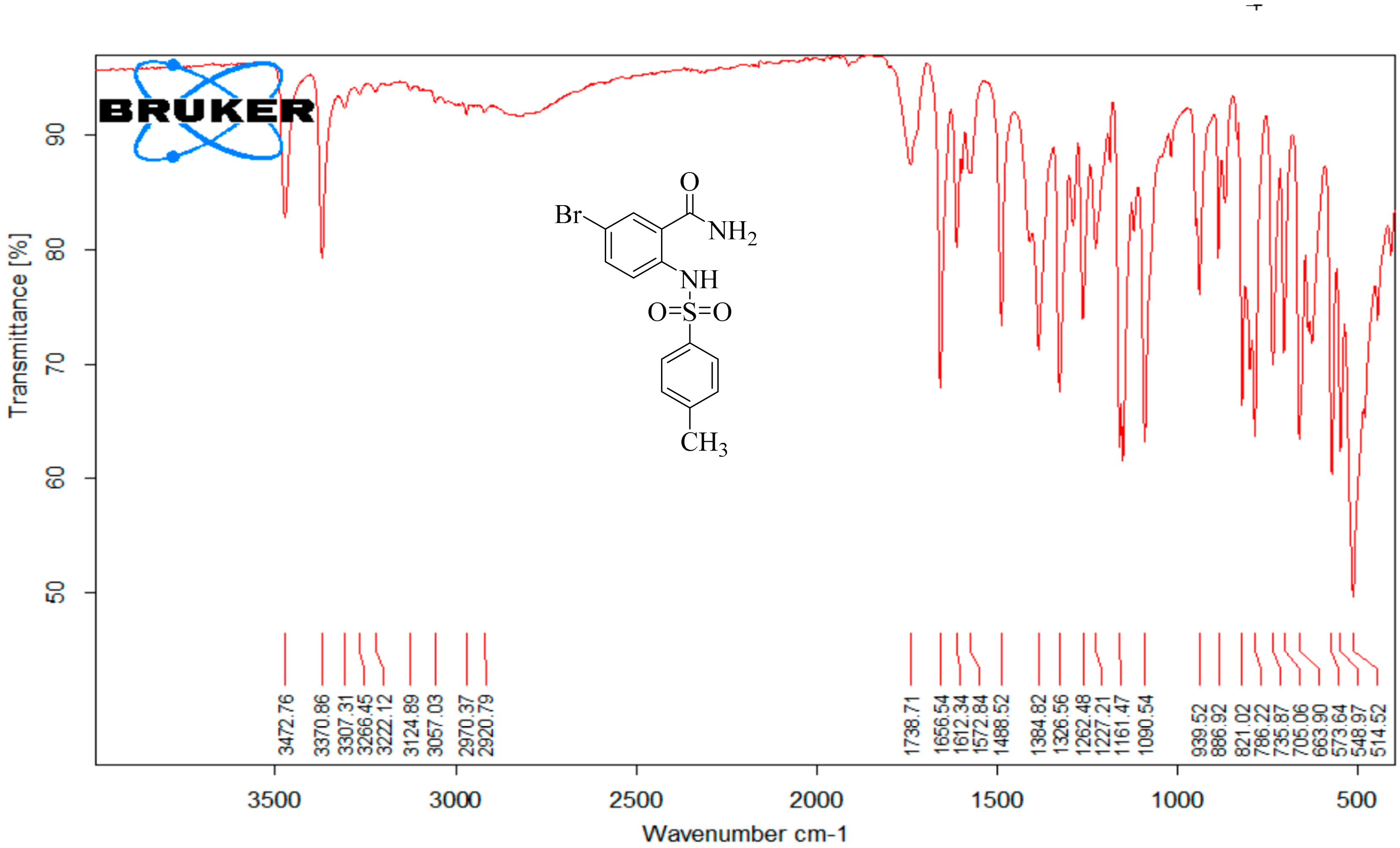

Figure S2.1: FT-IR spectrum of **2b**.

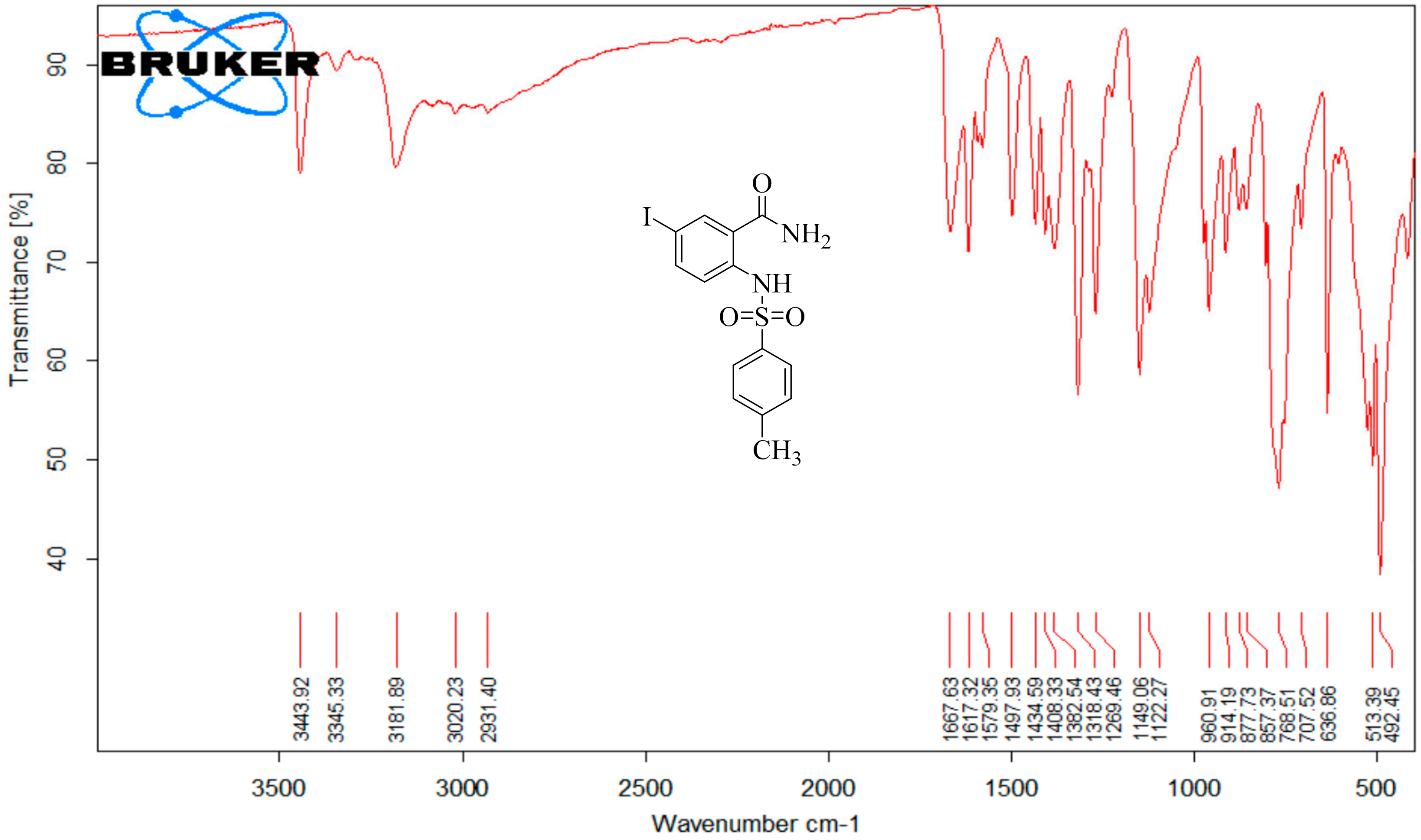

Figure S2.1: FT-IR spectrum of **2c**.

Table S1: Computed IR spectral data of **2a**, **2b** and **2c**.

2a		2b		2c	
**IR**	**INTENSITY**	**IR**	**INTENSITY**	**IR**	**INTENSITY**
16.6503	0.0171	15.6081	0.4407	11.897	0.9722
22.5698	0.6021	19.214	0.05	16.6306	1.0981
32.7304	0.1791	24.3875	0.1071	35.7279	0.4102
56.0339	0.2413	36.441	0.2429	45.5446	0.4126
69.6472	2.118	62.7791	0.1976	54.6408	8.6648
90.9853	3.4253	70.6164	4.6192	64.7766	0.1352
109.1577	1.3412	99.8242	4.1258	90.8698	2.1753
141.841	5.8515	113.1928	1.3858	112.5634	3.4203
159.0379	0.6379	144.5358	3.3374	134.7768	0.5731
185.5555	1.2513	152.5943	4.5254	138.385	4.0204
224.5497	8.6354	160.6522	0.5107	145.186	0.4875
262.1074	3.5033	201.3725	2.408	176.5431	0.3191
278.6141	0.0824	230.778	0.9191	189.5808	1.4618
285.0716	0.8796	249.8544	3.311	212.1788	4.0751
309.7716	9.3155	275.8041	3.6425	240.6837	12.479
334.9473	3.5693	311.3308	1.7851	279.4195	4.0848
359.8472	1.1087	320.5652	12.3922	299.0083	23.2149
376.9165	191.8061	333.6043	16.3163	318.946	7.2108
402.3468	29.3706	352.7371	8.038	323.9969	4.2028
413.8143	0.2514	368.29	15.1307	335.236	7.0903
421.492	3.1391	375.388	161.4816	391.3089	4.398
434.3434	0.094	402.9309	19.0627	405.0367	19.0059
476.073	5.5253	413.723	0.1386	411.3766	14.9529
512.9436	6.9209	424.6514	3.2294	418.7531	4.0905
527.4476	35.9276	442.8594	1.4168	422.3127	0.7311
543.8215	30.0201	476.6593	5.9854	451.9385	124.2753
564.3579	102.2483	518.7801	10.8383	499.7026	115.4018
568.3794	17.0737	530.317	58.6602	519.5828	15.831
617.1478	11.2389	546.8435	44.2849	535.898	43.577
639.6341	31.0302	572.0184	41.6074	552.6631	35.3172
648.3127	0.7509	579.2436	46.4549	601.2677	7.4673
650.2716	52.5319	632.9057	22.7757	611.9122	197.6608
712.0746	26.2695	648.0323	0.9246	617.3823	33.2143
716.0208	52.0621	655.6017	68.3795	643.6816	1.174
729.6337	79.7752	664.212	29.2689	647.3118	89.7275
761.289	78.5595	706.67	77.6829	718.5158	46.7173

773.7621	130.8163	714.9623	28.955	736.5404	1.9391
811.891	1.8607	727.8175	122.2382	739.0972	30.3334
814.7584	20.8791	787.0871	114.6803	772.744	50.2914
824.0668	14.384	806.041	14.4439	780.5336	49.1659
850.797	1.5134	813.7889	24.7352	795.8222	36.374
860.0073	23.473	823.2283	9.4974	811.3164	42.4329
892.5325	24.2451	846.3042	80.0447	831.8633	216.0859
910.9702	100.5823	849.6273	2.0808	869.2723	24.1828
966.96	0.2234	865.8025	16.8582	877.7691	66.0609
971.2337	3.7179	904.2658	60.7926	882.8536	26.3305
984.3865	0.0743	909.8785	14.4676	888.9947	1.8521
1006.1765	2.6762	966.6632	0.1635	918.2265	4.0651
1008.6147	0.6384	984.0921	0.0441	950.6523	54.6426
1033.2579	8.5674	992.0832	5.5837	1007.1022	1.2738
1062.203	12.5611	1009.645	0.35	1017.7967	1.5639
1069.7766	5.0489	1032.6401	8.6751	1022.0281	3.8311
1083.2554	96.9112	1061.9683	14.2011	1027.7437	2.6
1088.6305	6.7979	1082.3656	105.2035	1030.6321	1.6848
1117.7096	12.7522	1089.5512	7.602	1077.5108	6.9802
1132.4529	218.6192	1109.1997	55.4572	1082.4375	30.7427
1139.3995	38.3998	1118.7656	3.8679	1089.6023	14.398
1170.1656	1.7025	1132.9228	199.0697	1101.8258	20.0736
1192.2417	2.5114	1139.7675	40.9618	1131.4399	9.3109
1207.2414	4.815	1178.6431	3.3673	1154.1673	6.2001
1228.6708	2.9214	1207.2231	5.4756	1194.322	0.3345
1248.5658	21.5558	1228.9326	3.5671	1222.4561	12.9352
1291.303	140.0178	1251.4102	26.7487	1244.0787	6.4491
1320.5847	31.8909	1288.0022	147.7806	1272.043	5.3782
1326.4557	40.3401	1307.7723	9.5716	1327.4244	383.7526
1331.2514	16.0666	1324.7866	75.0719	1344.6182	34.513
1341.2975	53.144	1330.5988	12.0789	1345.3036	20.3268
1381.8086	168.686	1340.322	54.505	1377.0336	4.5941
1415.9749	1.3537	1380.4775	168.9991	1397.8001	86.325
1430.0641	5.1764	1415.5427	1.5048	1413.3699	106.581
1448.3861	106.8292	1420.716	80.6292	1431.5397	3.6722
1481.8813	21.2496	1430.3939	9.8081	1433.3262	191.0673
1490.3023	8.0842	1453.4988	43.1666	1448.778	12.9667
1495.1157	14.9696	1490.7591	9.0703	1515.4417	28.648
1524.5927	21.3346	1493.9113	13.5212	1515.9979	255.4956
1528.3929	132.0541	1514.0696	266.127	1518.6411	60.7343
1612.8546	39.2404	1524.0894	8.9324	1521.0151	12.6064

1615.3173	35.8362	1599.9499	30.6078	1592.0688	71.6097
1617.8371	75.1273	1614.1143	1.3203	1595.0056	55.9287
1639.1775	16.0106	1616.9261	79.9856	1631.8361	0.1181
1645.799	58.6298	1633.9598	64.9093	1639.2615	13.117
1723.5125	283.0648	1638.7007	20.1331	1648.6821	110.5528
3026.3298	23.155	1727.2942	278.4635	1676.7175	314.719
3080.3314	15.917	3026.2177	21.7996	3042.9924	26.4442
3108.0632	14.5457	3081.349	15.4588	3116.8564	18.9414
3165.6154	12.2923	3108.5829	13.7791	3146.8099	18.5442
3168.3365	11.501	3166.0707	12.0576	3202.4273	11.6407
3170.858	0.1689	3168.0397	10.2673	3208.3522	10.0559
3179.3197	13.5247	3198.2983	1.065	3223.571	8.4692
3194.926	16.3268	3200.8315	1.1061	3225.0495	1.367
3199.5937	1.0186	3203.2525	0.5124	3231.3298	2.4991
3208.8139	1.3307	3207.7586	1.2409	3237.3774	5.2448
3225.0327	3.5198	3225.5515	4.9881	3246.4928	3.7591
3365.309	256.4266	3371.6536	250.0708	3282.2499	423.8784
3591.3332	48.5467	3591.3199	52.9817	3621.5415	79.9027
3718.7202	35.7586	3718.3812	38.6458	3773.1897	38.0306

Table S2. Crystal data and structure refinement for compounds **2a–c.**

	**2a**	**2b**	**2c**
CCDC Number	2043901	2049537	2049356
Formula	$C_{14}H_{14}N_2O_3S$	$C_{14}H_{13}BrN_2O_3S$	$C_{14}H_{13}IN_2O_3S$
Formula weight	290.33	369.23	416.22
Temperature/K	173(2)	173(2)	173(2)
Wavelength/Å	0.71073	0.71073	0.71073
Crystal system	Monoclinic	Monoclinic	Monoclinic
Space group	P21/n	P21/n	C2/c
a/Å	8.7027(2)	7.7545(2)	22.966(3)
b/Å	11.6122(2)	14.1310(4)	5.0847(7)
c/Å	14.0951(3)	13.8992(4)	26.010(4)
α/°	90	90	90
β/°	104.4720(10)	00.7160(10)	103.560(5)
γ/°	90	90	90
V/Å^3	1379.22(5)	1496.50(7)	2952.7(7)
Z	4	4	8
d_{calc} (g.cm^{-3})	1.398	1.639	1.873
T_{min}, T_{max}	0.7029, 0.7457	0.5062, 0.7457	0.6461, 0.7457
Absorption coefficient (mm^{-1})	0.243	2.897	2.321
F(000)	608	744	1632
Crystal size (mm^3)	0.591 x 0.543 x 0.164	0.372 x 0.268 x 0.210 mm^3	0.558 x 0.120 x 0.024
Θ range for data collection	2.303 to 27.992°	2.074 to 27.997°	3.223 to 25.498°.
Index ranges	-11<=h<=11, -15<=k<=15, -18<=l<=18	-10<=h<=9, -17<=k<=18, -18<=l<=15	-27<=h<=27,6<=k<=6, -31<=l<=31
Reflections collected	22183	31198	56730
Independent reflections, R_{int}	3333, 0.0234	3609, 0.0217	2723, 0.0231
Data/restraints/parameters	3333 / 0 / 194	3609 / 0 / 203	2723 / 0 / 203
Goodness of fit on F^2	1.022	1.055	1.129
Final R indices [$I > 2s(I)$]	R1 = 0.0314, wR2 = 0.0855	R1 = 0.0222, wR2 = 0.0576	R1 = 0.0138, wR2 = 0.0355
R indices (all data)	R1 = 0.0346, wR2 = 0.0882	R1 = 0.0254, wR2 = 0.0591	R1 = 0.0141, wR2 = 0.0358

Figure S3: The Hirshfeld surface and 2D fingerprint plot of **2b** and **2c**.

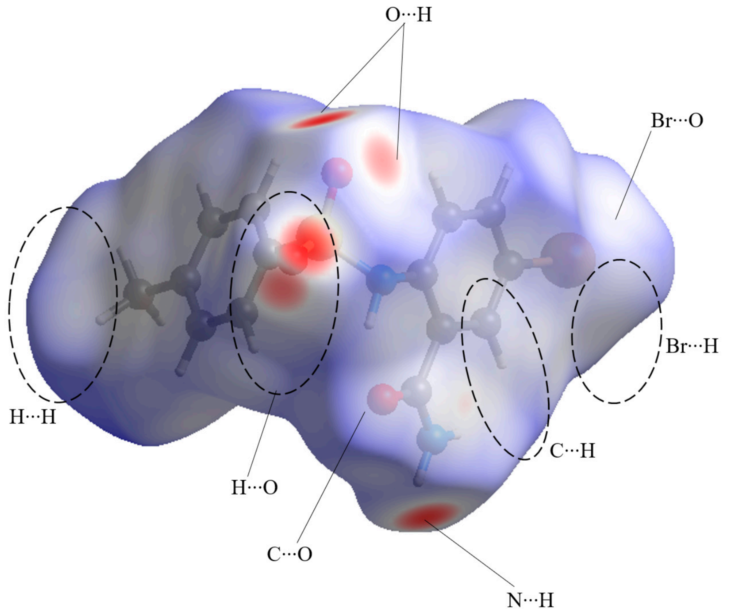

(a)

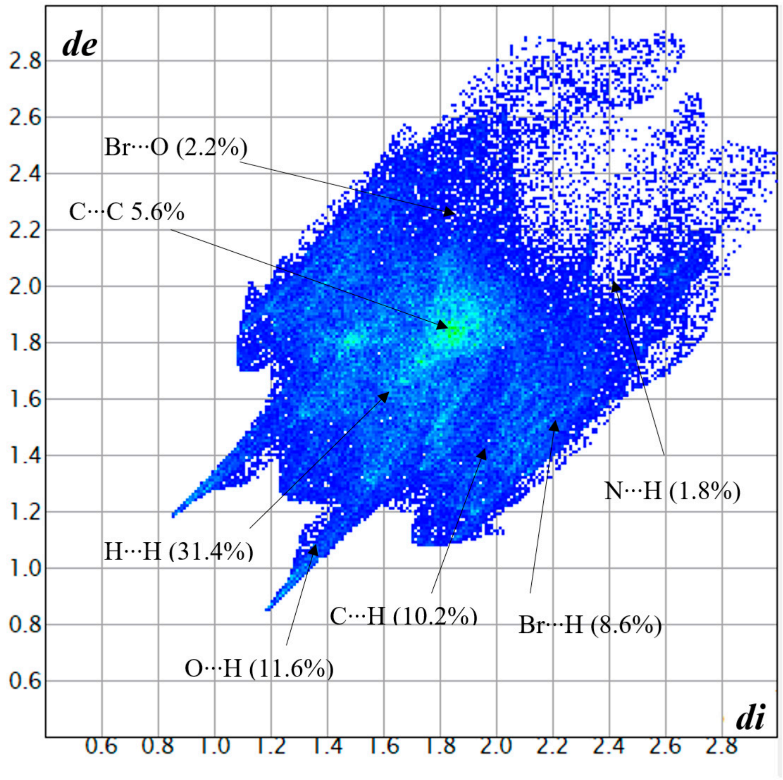

(b)

Figure S3.1: The Hirshfeld surface (a) and 2D fingerprint plot (b) of **2b.**

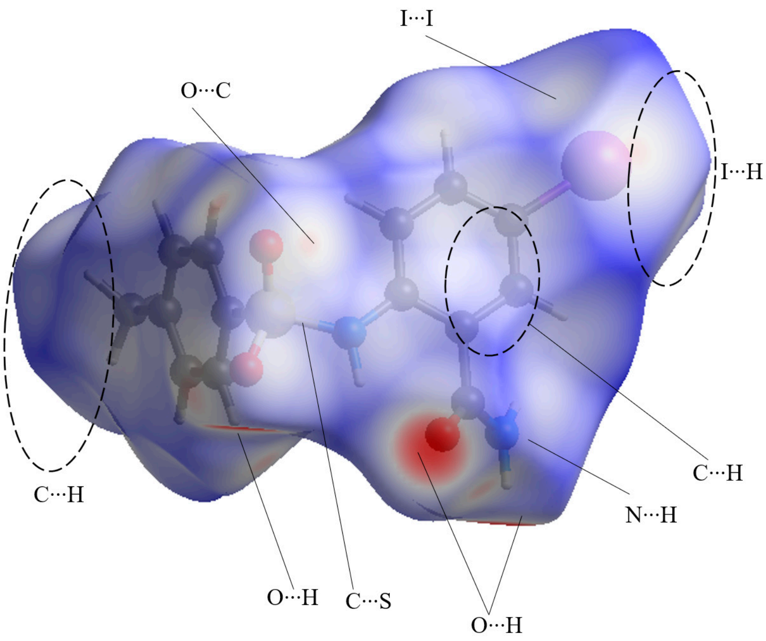

(a)

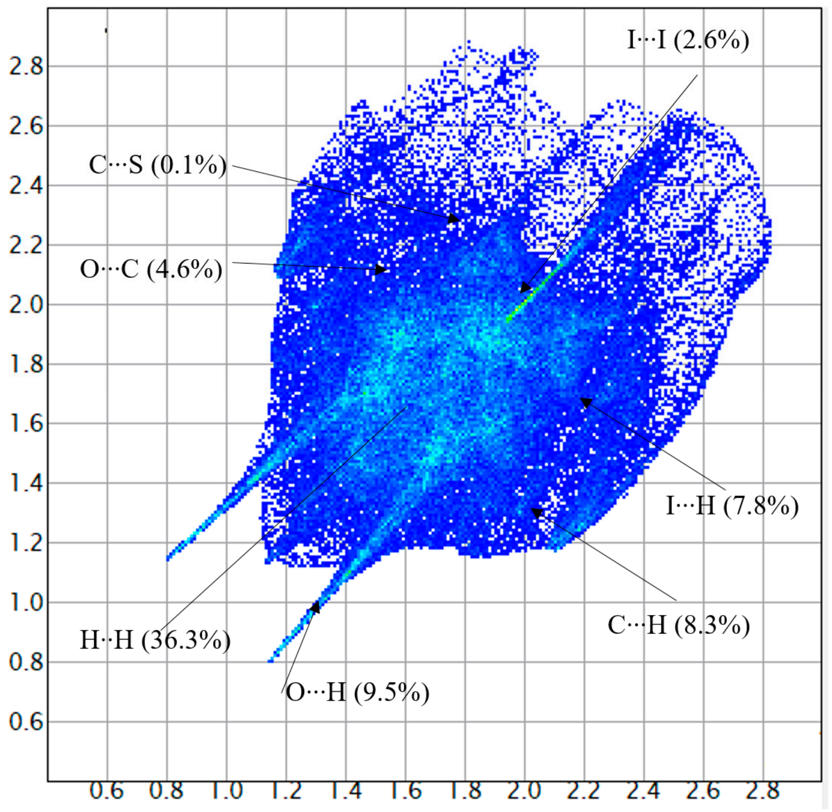

(b)

Figure S3.2: The Hirshfeld surface (a) and 2D fingerprint plot (b) of **2c**.

Table S3: Calculated bond lengths and bond angles of **2a, 2b** and **2c.**

2a		2b		2c	
R(1-2)	1.419	R(1-2)	1.418	R(1-2)	1.436
R(1-8)	1.404	R(1-8)	1.403	R(1-8)	1.416
R(1-10)	1.496	R(1-10)	1.498	R(1-10)	1.494
R(2-3)	1.403	R(2-3)	1.404	R(2-3)	1.420
R(2-30)	1.406	R(2-30)	1.403	R(2-30)	1.395
R(3-4)	1.081	R(3-4)	1.081	R(3-4)	1.084
R(3-5)	1.387	R(3-5)	1.386	R(3-5)	1.398
R(5-6)	1.084	R(5-6)	1.082	R(5-6)	1.086
R(5-7)	1.395	R(5-7)	1.393	R(5-7)	1.410
R(7-8)	1.386	R(7-8)	1.384	R(7-8)	1.397
R(7-34)	1.083	R(7-34)	1.916	R(7-34)	2.140
R(8-9)	1.084	R(8-9)	1.082	R(8-9)	1.085
R(10-25)	1.230	R(10-25)	1.229	R(10-25)	1.274
R(10-29)	1.367	R(10-29)	1.365	R(10-29)	1.374
R(11-12)	1.395	R(11-12)	1.395	R(11-12)	1.401
R(11-19)	1.391	R(11-19)	1.392	R(11-19)	1.397
R(11-28)	1.798	R(11-28)	1.797	R(11-28)	1.872
R(12-13)	1.083	R(12-13)	1.083	R(12-13)	1.086
R(12-14)	1.389	R(12-14)	1.389	R(12-14)	1.404
R(14-15)	1.085	R(14-15)	1.085	R(14-15)	1.088
R(14-16)	1.402	R(14-16)	1.401	R(14-16)	1.416
R(16-17)	1.398	R(16-17)	1.398	R(16-17)	1.412
R(16-21)	1.509	R(16-21)	1.508	R(16-21)	1.517
R(17-18)	1.085	R(17-18)	1.085	R(17-18)	1.087
R(17-19)	1.393	R(17-19)	1.392	R(17-19)	1.408
R(19-20)	1.082	R(19-20)	1.082	R(19-20)	1.086
R(21-22)	1.095	R(21-22)	1.095	R(21-22)	1.099
R(21-23)	1.092	R(21-23)	1.092	R(21-23)	1.095
R(21-24)	1.093	R(21-24)	1.093	R(21-24)	1.098
R(26-28)	1.455	R(26-28)	1.454	R(26-28)	1.624
R(27-28)	1.458	R(27-28)	1.458	R(27-28)	1.628
R(28-30)	1.689	R(28-30)	1.692	R(28-30)	1.784
R(29-31)	1.008	R(29-31)	1.008	R(29-31)	1.012
R(29-32)	1.006	R(29-32)	1.006	R(29-32)	1.008
R(30-33)	1.024	R(30-33)	1.024	R(30-33)	1.036
R(25-33)	1.825	R(25-33)	1.829	R(25-33)	1.752
A(2-1-8)	118.7	A(2-1-8)	119.1	A(2-1-8)	119.0

A(2-1-10)	120.8	A(2-1-10)	120.8	A(2-1-10)	120.2
A(1-2-3)	119.2	A(1-2-3)	118.9	A(1-2-3)	118.7
A(1-2-30)	120.1	A(1-2-30)	120.2	A(1-2-30)	118.9
A(8-1-10)	120.6	A(8-1-10)	120.1	A(8-1-10)	120.8
A(1-8-7)	121.7	A(1-8-7)	120.7	A(1-8-7)	121.3
A(1-8-9)	119.3	A(1-8-9)	120.0	A(1-8-9)	119.9
A(1-1025)	123.2	A(1-10-25)	122.8	A(1-10-25)	122.0
A(1-10-29)	116.7	A(1-10-29)	116.7	A(1-10-29)	118.9
A(3-2-30)	120.7	A(3-2-30)	120.8	A(3-2-30)	122.3
A(2-3-4)	118.6	A(2-3-4)	118.7	A(2-3-4)	119.4
A(2-3-5)	120.6	A(2-3-5)	121.1	A(2-3-5)	121.0
A(2-30-28)	125.5	A(2-30-28)	123.7	A(2-30-28)	129.1
A(2-30-33)	112.8	A(2-30-33)	113.0	A(2-30-33)	117.0
A(4-3-5)	120.8	A(4-3-5)	120.1	A(4-3-5)	119.5
A(3-5-6)	119.2	A(3-5-6)	119.9	A(3-5-6)	119.2
A(3-5-7)	120.7	A(3-5-7)	119.6	A(3-5-7)	120.3
A(6-5-7)	120.1	A(6-5-7)	120.4	A(6-5-7)	120.5
A(5-7-8)	119.1	A(5-7-8)	120.4	A(5-7-8)	119.7
A(5-7-34)	120.7	A(5-7-34)	119.9	A(5-7-34)	120.2
A(8-7-34)	120.2	A(8-7-34)	119.7	A(8-7-34)	120.2
A(7-8-9)	119.0	A(7-8-9)	119.2	A(7-8-9)	118.7
A(25-10-29)	120.2	A(25-10-29)	120.5	A(25-10-29)	119.1
A(10-25-33)	102.5	A(10-25-33)	102.7	A(10-25-33)	105.8
A(10-29-31)	115.8	A(10-29-31)	116.0	A(10-29-31)	117.1
A(10-29-32)	121.3	A(10-29-32)	121.6	A(10-29-32)	123.8
A(12-11-19)	121.0	A(12-11-19)	121.0	A(12-11-19)	123.2
A(12-11-28)	119.1	A(12-11-28)	119.1	A(12-11-28)	117.9
A(11-12-13)	120.2	A(11-12-13)	120.2	A(11-12-13)	120.7
A(11-12-14)	119.1	A(11-12-14)	119.0	A(11-12-14)	117.8
A(19-11-28)	119.8	A(19-11-28)	119.9	A(19-11-28)	118.9
A(11-19-17)	119.1	A(11-19-17)	119.0	A(11-19-17)	117.9
A(11-19-20)	119.8	A(11-19-20)	119.9	A(11-19-20)	120.5
A(11-28-26)	108.2	A(11-28-26)	108.3	A(11-28-26)	108.4
A(11-28-27)	107.5	A(11-28-27)	107.6	A(11-28-27)	107.2
A(11-28-30)	106.5	A(11-28-30)	107.5	A(11-28-30)	107.2
A(13-12-14)	120.7	A(13-12-14)	120.7	A(13-12-14)	121.5
A(12-14-15)	119.2	A(12-14-15)	119.2	A(12-14-15)	119.3
A(12-14-16)	121.2	A(12-14-16)	121.2	A(12-14-16)	121.2
A(15-14-16)	119.5	A(15-14-16)	119.5	A(15-14-16)	119.5
A(14-16-17)	118.4	A(14-16-17)	118.4	A(14-16-17)	118.7

A(14-16-21)	120.6	A(14-16-21)	120.6	A(14-16-21)	120.3
A(17-16-21)	121.0	A(17-16-21)	121.0	A(17-16-21)	121.0
A(16-17-18)	119.6	A(16-17-18)	119.6	A(16-17-18)	119.6
A(16-17-19)	121.2	A(16-17-19)	121.2	A(16-17-19)	121.2
A(16-21-22)	110.7	A(16-21-22)	110.7	A(16-21-22)	110.9
A(16-21-23)	111.5	A(16-21-23)	111.5	A(16-21-23)	111.6
A(16-21-24)	111.4	A(16-21-24)	111.4	A(16-21-24)	111.2
A(18-17-19)	119.2	A(18-17-19)	119.2	A(18-17-19)	119.2
A(17-19-20)	121.1	A(17-19-20)	121.0	A(17-19-20)	121.6
A(22-21-23)	107.6	A(22-21-23)	107.5	A(22-21-23)	107.7
A(22-21-24)	107.3	A(22-21-24)	107.3	A(22-21-24)	107.2
A(23-21-24)	108.2	A(23-21-24)	108.3	A(23-21-24)	108.0
A(26-28-27)	122.2	A(26-28-27)	122.3	A(26-28-27)	122.3
A(26-28-30)	104.0	A(26-28-30)	103.9	A(26-28-30)	102.4
A(27-28-30)	108.2	A(27-28-30)	108.0	A(27-28-30)	110.2
A(28-30-33)	112.1	A(28-30-33)	112.3	A(28-30-33)	114.9
A(31-29-32)	117.7	A(31-29-32)	117.8	A(31-29-32)	119.0
A(30-33-25)	137.5	A(30-33-25)	137.2	A(30-33-25)	134.3

Table S4: Second-order perturbation theory analysis of Fock matrix in NBO basis of **2a–c.**

Donor (i)	Type	ED/e	Acceptor (j)	Type	ED/e	$E^{(2)}$/kJmol^{-1}
			Compound **2a**			
C_1-C_2	π	-0.26354	C_2-N_{26}	σ*	0.39799	1.31
C_1-C_8	σ	-0.70228	C_{10}-O_{27}	π*	0.52485	2.06
C_2-N_{26}	σ	-0.80317	N_{26}-H_{34}	σ*	0.44532	0.73
C_{10}-O_{27}	π	-0.39271	N_{26}-H_{34}	σ*	0.44532	0.84
C_{10}-N_{25}	σ	-0.85157	N_{25}-H_{33}	σ*	0.47803	0.53
O_{28}-S_{30}	σ	-0.97846	N_{26}-H_{34}	σ*	0.44532	0.75
S_{30}-H_{31}	σ	-0.76841	O_{29}-S_{30}	σ*	0.26594	1.36
N_{25}-H_{33}	σ	-0.67517	C_{10}-O_{27}	π*	0.52485	1.23
N_{26}-H_{34}	σ	-0.65955	O_{29}-S_{30}	σ*	0.26765	0.91
LP(1)N_{26}	σ	-0.27315	O_{28}-S_{30}	σ*	0.26594	3.74
LP(1)N_{25}	σ	-0.31783	C_{10}-O_{27}	π*	0.52485	58.16
LP(1)O_{27}	σ	-0.68102	N_{26}-H_{34}	σ*	0.44532	5.52
LP(2)O_{27}	*n*	-0.28269	N_{26}-H_{34}	σ*	0.44532	12.49
LP(1)O_{28}	σ	-0.79290	O_{29}-S_{30}	σ*	0.26594	1.50
LP(2)O_{28}	*n*	-0.29832	S_{30}-N_{26}	σ*	0.12808	10.12
LP(3)O_{28}	*n*	-0.29738	S_{30}-N_{26}	σ*	0.12808	12.49
LP(1)O_{29}	σ	-0.79362	S_{30}-N_{26}	σ*	0.12808	1.43
LP(2)O_{29}	*n*	-0.30037	S_{30}-N_{26}	σ*	0.12808	10.84
LP(3)O_{29}	*n*	-0.29858	S_{30}-N_{26}	σ*	0.12808	13.54
			Compound **2b**			
C_1-C_2	π	-0.26920	N_{26} - S_{30}	σ*	0.11742	2.70
C_1-C_8	σ	-0.71219	N_{26} - H_{34}	σ*	0.44751	1.51
C_1-N_{26}	σ	-0.82167	O_{29}-S_{30}	σ*	0.27202	0.58
C_{10}-N_{25}	σ	-0.83946	C_{10}-O_{27}	π*	0.01295	0.57
C_{10}-O_{27}	π	-0.39922	N_{25}-H_{33}	σ*	0.47895	0.70
N_{26}-S_{30}	σ	-0.76939	O_{29}-S_{30}	σ*	0.27202	3.26
N_{26}-H_{34}	σ	-0.66725	O_{29}-S_{30}	σ*	0.27202	0.75
N_{25}-H_{33}	σ	-0.65971	C_{10}-O_{27}	π*	0.01295	1.61
O_{28}-S_{30}	σ	-0.98940	O_{29}-S_{30}	σ*	0.27202	1.87
O_{29}-S_{30}	σ	-0.99272	N_{26}-S_{30}	σ*	0.11742	2.03
LP(1)N_{25}	σ	-0.28081	C_{10}-O_{27}	π*	0.01295	51.40
LP(1)N_{26}	σ	-0.30961	C_1-C_2	π*	0.00604	22.25
LP(1)O_{27}	σ	-0.68632	N_{26}-H_{34}	σ*	0.44751	5.06
LP(2)O_{27}	*n*	-0.28156	C_{10}-N_{25}	σ*	0.45590	22.85
LP(1)O_{28}	σ	-0.79731	O_{29}-S_{30}	σ*	0.27202	1.75
LP(2)O_{28}	*n*	-0.30692	O_{29}-S_{30}	σ*	0.27202	2.83
LP(3)O_{28}	*n*	-0.30551	O_{29}-S_{30}	σ*	0.27202	19.44
LP(1)O_{29}	σ	-0.79511	N_{26}-S_{30}	σ*	0.26646	1.81
LP(2)O_{29}	*n*	-0.30493	O_{28}-S_{30}	σ*	0.26646	2.75

LP(3)O_{29}	*n*	-0.30393	O_{28}-S_{30}	σ*	0.26646	20.30
LP(1)Br_{31}	σ	-0.96161	C_3-C_5	σ*	0.56488	1.18
LP(2)Br_{31}	*n*	-0.30160	C_3-C_5	σ*	0.56488	2.97
LP(3)Br_{31}	*n*	-0.30108	C_3-C_5	π*	0.00408	9.46
			Compound **2c**			
C_1-C_2	π	-0.27423	C_{10}-O_{27}	σ*	0.02103	27.32
C_1-C_8	σ	-0.71168	N_{26}-H_{34}	σ*	0.44275	2.60
C_1-N_{26}	σ	-0.84401	O_{29}-S_{30}	σ*	0.08292	0.57
C_{10}-N_{25}	σ	-0.85536	C_2-C_{10}	σ*	0.39065	0.61
C_{10}-O_{27}	π	-0.38877	N_{25}-H_{33}	σ*	0.43820	2.25
N_{26}-S_{30}	σ	-0.71458	O_{29}-S_{30}	σ*	0.08292	2.09
N_{26}-H_{34}	σ	-0.67974	O_{29}-S_{30}	σ*	0.08292	0.99
N_{25}-H_{33}	σ	-0.70688	C_{10}-O_{27}	σ*	0.02103	3.61
O_{28}-S_{30}	σ	-0.75356	O_{29}-S_{30}	σ*	0.08292	2.49
O_{29}-S_{30}	σ	-0.75549	N_{26}-S_{30}	σ*	0.05324	2.28
LP(1)N_{25}	σ	-0.28939	C_{10}-O_{27}	π*	-0.03137	61.93
LP(1)N_{26}	σ	-0.28455	C_1-C_2	π*	-0.00132	38.78
LP(1)O_{27}	σ	-0.68319	N_{26}-S_{30}	σ*	0.05324	5.04
LP(2)O_{27}	*n*	-0.30507	C_{10}-N_{25}	σ*	0.39649	19.61
LP(1)O_{28}	σ	-0.90840	O_{29}-S_{30}	σ*	0.08292	1.72
LP(2)O_{28}	*n*	-0.28621	N_{26}-S_{30}	σ*	0.05324	3.79
LP(3)O_{28}	*n*	0.28373	O_{29}-S_{30}	σ*	0.08292	11.91
LP(1)O_{29}	σ	-0.90747	N_{26}-S_{30}	σ*	0.05324	2.28
LP(2)O_{29}	*n*	-0.29466	N_{26}-S_{30}	σ*	0.05324	3.65
LP(3)O_{29}	*n*	-0.28752	O_{29}-S_{30}	σ*	0.08292	10.79
LP(1)I_{31}	σ	-0.60356	C_5-C_6	σ*	0.50498	1.29
LP(2)I_{31}	*n*	-0.27417	C_5-C_6	σ*	0.50498	2.67
LP(3)I_{31}	*n*	-0.27157	C_3-C_5	π*	-0.00188	6.72
